# Satisfactory breeding potential is transiently eliminated in beef bulls with clinical anaplasmosis

**DOI:** 10.1186/s12917-022-03470-7

**Published:** 2022-10-29

**Authors:** Anne C. Lovett, Emily J. Reppert, John R. Jaeger, Qing Kang, Macy R. Flowers, Naemi P. Bickmeier, Tippawan Anantatat, Shannon C. O’Day, Chance L. Armstrong, Kathryn E. Reif

**Affiliations:** 1 Diagnostic Medicine/Pathobiology, Kansas State University, 66506 Manhattan, KS USA; 2 Clinical Sciences, Kansas State University, 66506 Manhattan, KS USA; 3 Western Kansas Agricultural Research and Extension Center, Kansas State University, 67601 Hays, KS USA; 4 Statistics, Kansas State University, 66506 Manhattan, KS USA; 5 Veterinary Clinical Sciences, Louisiana State University, 70803 Baton Rouge, LA USA

**Keywords:** *Anaplasma marginale*, Anemia, Breeding Soundness Examination (BSE), Cattle, Fever, Reproduction, Scrotal circumference, Sperm morphology, Sperm motility, Veterinary theriogenology

## Abstract

**Background:**

Natural service breeding is common in U.S. cow-calf operations. Diseases impacting bull reproductive performance have significant economic consequences for producers. Anaplasmosis may be an underappreciated cause of poor reproductive performance in bulls. The primary systemic effects of bovine anaplasmosis including anemia, fever, and weight loss, can all result in unsatisfactory reproductive performance. The objective of this pilot study was to evaluate breeding soundness examination (BSE) outcomes and clinical changes in bulls during and upon resolution of clinical anaplasmosis. *Anaplasma marginale*-challenged bulls were observed for clinical disease and infection progression and changes in breeding soundness compared to uninfected control bulls for 16 weeks.

**Results:**

All *Anaplasma marginale*-challenged bulls were PCR-positive, seropositive, and showed clinical signs by 3-, 17-, and 24-days post-challenge, respectively. Clinical signs of anaplasmosis included pallor, icterus, fever (≥ 40.2 °C), and weight loss. Acute anemia was observed in all challenged bulls with PCV nadirs ≤ 18% and peak percent parasitized erythrocyte ≥ 50%. Decreased scrotal circumference and poor semen quality (e.g., increased percentage of abnormal spermatozoa, decreased progressively motile sperm), were initially observed within days after onset of clinical anaplasmosis signs and continued weeks beyond disease resolution. Control bulls remained negative for *A. marginale*.

**Conclusion:**

This pilot study demonstrates that clinical anaplasmosis reduces breeding soundness in beef bulls. Anaplasmosis should be considered as a differential for bulls with decreased semen quality, especially within endemic areas. A 90 day or greater retest window is recommended for bulls of unsatisfactory breeding potential recently recovered from clinical anaplasmosis.

**Supplementary Information:**

The online version contains supplementary material available at 10.1186/s12917-022-03470-7.

## Background

Bull fertility is a key component to success and profitability in commercial cow-calf operations. In July 2021, the United States Department of Agriculture’s National Agricultural Statistics Services reported an estimated 2.1 million beef bulls in the United States (U.S.) and 40.9 million beef cows of calving age [1]. In the U.S., 92.9% of commercial beef cows are naturally bred by bulls [2, 3], highlighting the importance of sound bull reproductive performance to the cow-calf industry. On average, an individual commercial Angus bull over 3 years of age in the U.S. costs $2,500 - 5,000 and is expected to service up to 60 head of cows and heifers in a breeding season. Fertile bulls positively contribute to a herd through a variety of avenues including exceptional genetics, calf crops, and annual profits [4, 5]. Investing in routine breeding soundness examinations (BSEs) is one way to assess breeding potential in bulls and improve chances for breeding success [6], with a potential benefit-to-cost ratio of approximately 7 for every dollar spent on a BSE [7].

Fertility is five times more valuable than production traits such as beef merit or average daily gain in a beef cow-calf herd [8]. Subfertile bulls are detrimental to a cow-calf producer’s profitability, from feed costs of non-productive animals to a prolonged calving season [9–11]. Some diseases can negatively affect bull reproductive parameters and fertility, causing an adverse chain reaction for herd and producer success, often going unnoticed until assessment of cow pregnancy status [5, 12]. One disease with a potentially underappreciated, negative impact on bull breeding soundness is anaplasmosis, caused by the tick-borne rickettsial pathogen, *Anaplasma marginale*. Anaplasmosis in cattle causes anemia, fever, weight loss, lethargy, and death in severe acute cases [13–15]. Few direct studies have been performed assessing the impact of clinical anaplasmosis on beef bull fertility [15, 16]; but, anemia, fever, and weight loss are all associated with unsatisfactory breeding outcomes in bulls [2, 17, 18]. Anemia leads to exacerbated hypoxic conditions in testicular tissue, ultimately affecting spermatogenesis and thermoregulation [19–22]. Fever or increased body temperature increases the metabolic rate in tissues, and prolonged fever can result in testicular degeneration, impairments in spermatogenesis, and direct damage to sperm cells [2, 23–25]. Weight loss and lethargy negatively affect normal sperm production, sperm viability, and bull libido [8, 17, 26], all of which are essential for natural-service breeding bulls [9]. Finally, systemic illness can cause reductions in breeding soundness from a heightened inflammatory response and increased cortisol production [2, 27, 28].

The distribution of *A. marginale* is expansive, present in tropical, subtropical, and temperate regions worldwide [29, 30]. In the U.S., *A. marginale* has been reported in most continental states, and is most prevalent in the Gulf Coast, Midwest and Western regions—some of the highest cattle-producing regions in the country [13, 29, 30]. Statewide anaplasmosis seroprevalence studies demonstrate beef cattle seroprevalence rates of 4.4 – 29.0% [31–33]. Further, a 2016–2017 study in Kansas identified 52.5% of beef cattle herds seropositive for *A. marginale* [34]. The expansive distribution of anaplasmosis in endemic areas is in part facilitated by the pathogen establishing persistent infection in its primary reservoir host, cattle. Persistently infected (PI) carrier cattle serve as a reservoir and source of infection to naïve cattle through arthropod vector or iatrogenic transmission. Considering disease prevalence and PI carriers, a large proportion of bulls in the U.S. are at risk of exposure to *A. marginale* and developing clinical anaplasmosis. Therefore, the objective of this study was to determine the effects of acute *A. marginale* infection on overall breeding soundness examination (BSE) pass rate, including sperm morphology and progressive motility, and scrotal circumference in commercial beef bulls during and upon resolution of clinical anaplasmosis. We hypothesized that bulls with clinical anaplasmosis, including disease-associated anemia and fever, would have reduced semen quality and, subsequently, lower BSE pass rates. Information on how clinical anaplasmosis negatively affects bull breeding soundness will inform strategic anaplasmosis and herd management decisions, including BSE recommendations for bulls in anaplasmosis-endemic areas.

## Methods

### Study animals

Six crossbred beef bulls from the Kansas State University Western Kansas Agricultural Research and Extension Center herd (Hays, Kansas) were enrolled in this pilot study. Requirements for bull enrollment included: healthy on initial physical examination; ≥24-months of age; sound breeding potential (met minimum standards of a complete BSE on two separates dates within 3 months of study start date); negative for *A. marginale* via PCR; and negative for *A. marginale*-specific antibodies via serum cELISA testing. At the time of the study, bull weights ranged from 454 to 799 kg (mean = 619 kg, median = 562 kg). Bull ages ranged from 24- to 48-months old (median = 24-months old).

Bulls in the same treatment group were co-housed in an outdoor dry lot pen for approximately six months. During that time, bulls had *ad libitum* access to fresh water and were fed a standard ration of grain at 0.5% body weight per day with *ad libitum* access to hay.

As the bulls originated from a working herd of cattle, the potential for other common comorbidities in study bulls was evaluated. All bulls were tested for potential co-infection of Bluetongue virus (BTV), Bovine Leukemia Virus (BLV), and Bovine Viral Diarrhea Virus (BVDV). No bulls demonstrated clinical signs associated with any of these potential co-infections at study enrollment. At study enrollment, all bulls were seropositive by ELISA but PCR negative for BTV; bulls 8553, 9431, 9600, and 7532 were seropositive for BLV; and all bulls were negative for BVDV by antigen capture ELISA. Bulls that were subsequently challenged with *A. marginale* were re-tested for these possible co-infections at peak fever and all maintained their pre-study status for BTV, BLV and BVDV, indicating that these co-infections had minimal to no contribution on study outcomes. Furthermore, previous work has demonstrated that positive serologic status for these infections in bulls has not demonstrated unsatisfactory semen quality [35–37].

### Study Design

All animal studies were carried out under an approved Institutional Animal Care and Use protocol (IACUC 4476) and an approved Institutional Biosafety Committee protocol (IBC 1495) on file at the University Research Compliance Office at Kansas State University, Manhattan, KS. All methods were carried out in accordance with relevant guidelines and regulations in compliance with ARRIVE guidelines.

Enrolled bulls were blocked by weight and allocated randomly into two equally sized groups using the RAND function in a spread-sheet program (Excel, Microsoft Office, Richmond, WA). Three bulls (animal IDs: 8553, 9528, 9431) were included in the *A. marginale* challenge group (ANA) and were inoculated on Day 0 with blood from a persistently infected donor cow (*see ‘Anaplasma marginale infection challenge’*). The other three bulls (animal IDs: 7532, 9550, 9600) were included in the unchallenged control group (CON). Breeding soundness examinations were performed weekly on all bulls starting two weeks prior to Day 0 and weekly through Day 84. Two additional BSEs were performed on all bulls on Day 98 and Day 112, 14- and 16-weeks post-inoculation, respectively.

Blood samples were collected from all bulls (ANA and CON) once weekly for 3 weeks prior to inoculation; twice weekly post-inoculation (Day 0 to Day 41); once weekly from Day 42 to 84; and on Day 98 and 112. At each blood collection time point, approximately 20 mL of blood was obtained from the coccygeal vein and distributed between evacuated tubes (Vacutainer, Becton Dickinson, Franklin Lakes, NJ) containing EDTA (1.8 mg/mL whole blood) and tubes with no anticoagulant. Anemia was assessed by quantifying packed cell volume (PCV) from whole blood collected into EDTA tubes and then centrifuged in microhematocrit capillary tubes for 5 min (Micro-Hematocrit centrifuge CMH30, UNICO, Dayton, NJ). A bull was considered anemic if their PCV was reduced by ≥ 25% of their respective baseline value (baseline determined from the mean of three time points prior to inoculation) [38, 39]. A rectal temperature was obtained on each bull (Kroger® Comfort Flex Tip Digital Thermometer, Cincinnati, OH) at every blood sample time point. A bull was considered febrile if its rectal temperature was ≥ 39.2 °C, and hypothermic if its rectal temperature was ≤ 38.0 °C [38]. All bulls were monitored pen-side daily for signs of clinical anaplasmosis (e.g., tachypnea, anorexia, pallor, lethargy, and icterus) and overall health status. Animals were recorded as “healthy” unless clinical signs were noted.

Severe clinical anaplasmosis included a decrease in PCV of 7% or greater in < 48 h, a PCV ≤ 15%, fever (≥ 40.8 °C), and any combination of pale or icteric mucous membranes, lethargy, anorexia, or loss of body condition. If two or more of these clinical signs were appreciated on daily health checks or at time of blood draw, the bull was administered oxytetracycline subcutaneously (Bio-Mycin® 200, Boehringer Ingelheim Vetmedica Inc, Duluth, GA) at a dose rate of 20 mg/kg of body weight for up to 3 doses given once every 48 h. The number of oxytetracycline doses administered depended on an individual bull’s clinical status.

All bulls were treated every two weeks with 30 mL of a topical permethrin (Ultra Boss®, Merck Animal Health, NJ) applied across the topline to control pre-existing lice and for the control of any potential arthropod vectors.

### *Anaplasma marginale* infection challenge

The *A. marginale* inoculate used as challenge material was obtained from an apparently healthy, persistently infected, mature donor cow (animal ID: 6473) from the Kansas State University Western Kansas Agricultural Research and Extension Center Cow-Calf Unit in Hays, Kansas. The donor was confirmed positive for *A. marginale* via PCR prior to the beginning of the study. This cow was also seropositive but PCR negative for BTV and seropositive for BLV. To collect blood, the donor jugular site was clipped and aseptically prepped with povidone iodine scrub and alcohol. A 14-gauge needle was placed in the right jugular vein and attached to a blood collection set, and approximately 400 mL of blood was collected into a heparinized, 450-mL blood collection bag. The blood was placed on ice for transport and maintained at 4 °C until the time of inoculation the next morning. The three bulls in the ANA group were inoculated with 40 mL of donor cow whole blood by direct venipuncture into the right jugular vein. Bulls were monitored for 15 min after inoculation for any signs of hypersensitivity reaction or anaphylaxis.

The strain of *A. marginale* in the donor cow was identified by amplifying, cloning, and sequencing the variable region of the Major Surface Protein 1a gene to determine the Msp1a genotype(s) as previously described [40]. The *A. marginale* challenge strain was confirmed in each experimentally infected bull. Sequences from this study were submitted to GenBank and are available at the following accession numbers: ON597854-ON97859.

### Breeding soundness examination (BSE)

All BSEs were performed by the same veterinarian using the *Society for Theriogenology Manual for Breeding Soundness Examination of Bulls*, 2nd Edition guidelines [41]. To perform BSEs, bulls were restrained in a squeeze chute. Semen samples were collected via electroejaculation (Pulsator V, Lane Manufacturing Company, Denver, CO). As part of a routine, complete BSE, the following were evaluated and are described in detail below: general physical health including external and internal genitalia, scrotal circumference measurement, response to electroejaculation, sperm morphology, and sperm progressive motility.

General physical examination included an overall health assessment with specific evaluation of foot and leg soundness, evaluation of vision, palpation of external genitalia, and rectal palpation of internal genitalia. Body condition score (BCS) assessment was considered part of the general physical exam, and was assigned based on a 9-point scoring system [42]. All examination findings were reported on a standardized BSE form [41]. External genitalia assessment included palpation of penile sheath and prepuce, penis, scrotum and testes for any changes in texture. Abnormalities of the external genitalia, including changes in scrotal size and shape, testicular texture, or penile abrasions, were recorded on the examination form. Scrotal circumference was determined using a scrotal measuring tape (cm) around the widest girth of the scrotum, with a minimum of 34 cm required to pass based on the age of enrolled bulls. Internal genitalia assessment was performed by rectal palpation of seminal vesicles, ampullae, prostate, and inguinal rings. Abnormalities of the internal genitalia, including enlarged seminal vesicles, were recorded. Response to electroejaculation was based on presence or absence of protrusion, erection, and ejaculation.

Sperm morphology was evaluated from a minimum of 100 sperm cells via microscopy using eosin-nigrosin stain (Live/Dead Semen Stain, Jorvet, Loveland, CO) under 1,000X magnification in oil. Each sperm cell was categorized into “normal”, “head abnormality”, “midpiece abnormality”, or “tail abnormality,” with a minimum of 70% morphologically normal sperm cells required for passing. Examples of sperm head abnormalities include pyriform heads and nuclear vacuolations. Examples of sperm midpiece abnormalities include proximal droplets and distal midpiece reflections (DMRs). Examples of sperm tail abnormalities include coiled tails. Presence or absence of other abnormal cells in the ejaculate, such as white cells (i.e. neutrophils, macrophages) or immature sperm cells — spermatocytes — was assessed with Wright-Giemsa-stained semen smears (HEMA-3, Fisher HealthCare, Pittsburgh, PA) under 1,000X magnification in oil. Minimal to no white blood cells or spermatocytes in the semen were required for satisfactory breeding potential.

Sperm motility was assessed chute-side using a compound microscope at 100X magnification (Amscope, Irvine, CA), and scored on a percentage basis, with a minimum of 30% sperm moving in a forward linear motion per high powered field required to pass this metric. Warm, sterile, phosphate-buffered saline (PBS) was incorporated into a drop of neat semen to dilute the sample for more accurate evaluation of sperm cell progressive motility.

Collectively, bulls were considered of satisfactory breeding potential if they had: (i) no abnormalities on general physical examination; (ii) no abnormalities on assessment of internal and external genitalia; iii) ≥ 34 cm scrotal circumference; iv) ≥ 70% sperm cells of normal morphology; and v) ≥ 30% of sperm with progressive motility. Bulls were considered of unsatisfactory breeding potential if they did not meet minimum requirements in any of the above categories.

### DNA extraction and quantitative PCR (qPCR) for bacteremia determination

Total genomic DNA (gDNA) was extracted from 100 µL of whole blood collected into EDTA tubes using the Quick-DNA Miniprep Kit (Zymo Research, Irvine, CA) according to manufacturer instructions, and the resulting gDNA was eluted in 35 µL of DNA Elution Buffer. Genomic DNA was stored at -20 °C. A quantitative, real-time PCR (qPCR) assay targeting a portion of the single-copy *A. marginale* Msp5 gene [43] was used to quantify *A. marginale* bactermia. PCR mastermix preparations were set up in 20 µL reaction volumes, each containing 0.2 µM of each primer (*Am* msp5 F: ATA CCT GCC TTT CCC ATT GAT GAG GTA CAT and *Am* msp5R AGG CGA AGA AGC AGA CAT AAA GAG CGT), 10 µL of SsoAdvanced Universal SYBR Green Supermix (Bio-Rad, Hercules, CA), and 2 µL gDNA. Reaction cycling was performed using a CFX Connect Real-Time PCR System (Bio-Rad), using the following cycling parameters: one cycle of 98 °C for 2 min; followed by 40 cycles at 98 °C for 5 s, 60 °C for 5 s and 74 °C for 15 s; and a final melt curve cycle of 65 – 95 °C with increasing 0.5 °C temperature steps at 10 s/step. Real-time qPCR data was visualized and analyzed using CFX Maestro Software v1.1 (Bio-Rad).

### Percent parasitized erythrocyte (PPE) determination

The percentage of red blood cells infected with *A. marginale* was assessed via microscopic examination of Wright-Giemsa-stained blood smears (HEMA-3, Fisher HealthCare, Pittsburgh, PA). Total red blood cells in a monolayer were counted microscopically under 1,000X magnification. The number of *A. marginale*-infected red blood cells were divided by the total number of evaluated red blood cells, and that value multiplied by 100 to determine the PPE. A minimum of 200 red blood cells were evaluated per sample.

### Competitive ELISA

Blood samples collected into tubes without anticoagulant were centrifuged at 3,000 rpm for 10 min at 20 °C to separate and collect serum. Serum samples were aliquoted and stored at -80 °C before testing. Serum samples were submitted to the Iowa State University Veterinary Diagnostic Laboratory (Ames, IA) for *A. marginale* serological screening using a commercial cELISA that detects host antibody produced against *A. marginale* Msp5 (*Anaplasma* Antibody Test Kit, cELISA v2, VMRD, Pullman, WA). Animals with a percent inhibition score ≥ 30% were considered seropositive for *A. marginale*.

### Statistical analysis

Packed cell volume percent change and scrotal circumference percent change from baseline were analyzed separately at each post-challenge study day under the linear model with treatment being the fixed effect. Percent normal morphology and percent progressive motility were analyzed separately at each post-challenge study day, under the linear model with treatment being the fixed effect and baseline average being the covariate. All hypothesis tests were 2-sided tests. Statistical analysis was performed using Statistical Analysis Software (SAS 9.4; Cary, NC) MIXED procedure.

## Results

### Donor animal anaplasmosis characteristics

Blood from a healthy, 4-year-old, Angus-cross cow persistently infected with *A. marginale* was used to challenge ANA group bulls. The PCV and *A. marginale* bacteremia of the donor was 32% and 1.15E + 06 *A. marginale*/mL blood, respectively. The *A. marginale* Msp1a genotype identified in the donor cow was: A B B B.

### Progression of clinical anaplasmosis in experimentally-infected bulls

**Fig. 1:**
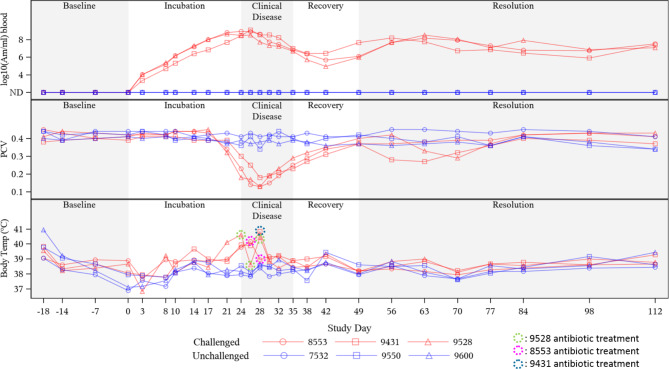
**Progression of clinical anaplasmosis in experimentally-challenged beef bulls.** Top: Progression of *A. marignale* bacteremia (*A. marginale* (*Am*)/mL blood) evaluated using a quantitative PCR assay targeting the single-copy Msp5 gene. Middle: Changes in packed cell volume (PCV) before, during, and after resolution of clinical anaplasmosis. Bottom: Changes in body temperatures (°C ) prior to, during, and after resolution of clinical anaplasmosis. Figure background shading represents phases of clinical anaplasmosis with Day 0 representing day of *A. marginale* inoculation in challenged bulls.

All ANA bulls developed clinical anaplasmosis and the infecting strain was confirmed to be Msp1a genotype A B B B. All *A. marginale* inoculated bulls developed anemia and fever. Time to initial signs of clinical anaplasmosis (i.e. anemia, fever) ranged from 21 to 24 days post-challenge (mean = 23 days, median = 24 days) (Fig. 1, Supplemental Table 1, Supplemental Table 2). Signs of clinical anaplasmosis lasted 16 to 17 days (mean = 16.7 days, median = 17 days).

Bull PCV was evaluated to track progression of anemia (Fig. 1, Supplemental Table 1). Packed cell volume nadirs were reached in all ANA bulls 28 days after inoculation and the average PCV nadir was 15% (median = 13%, range: 13 – 18%), and averaged 64.1% (median = 69.2%, range: 53.8 − 69.4%) loss of red blood cell from baseline (Table 1). All ANA bulls had pale or icteric mucous membranes including pallor of the prepuce during peak clinical disease (28 days after inoculation). Individual bull PCV loss compared to their respective baseline value was significant (*P* = < 0.001, Table 1). Compared to unchallenged, time-matched CON bulls which experienced no appreciable loss in PCV throughout the study, ANA bulls had significantly lower PCVs on Day 28 (54% lower; *P* = 0.002) through at least Day 42 (14 days beyond ANA PCV nadir). All bulls in the ANA group developed fevers (mean = 40.6 °C, median = 40.6 °C, range: 40.2 − 40.8 °C) which peaked 24 to 28 days after inoculation (Fig. 1, Supplemental Table 2). Fevers were intermittent around peak infection and lasted 7 to 8 days (mean = 7.3 days, median = 7 days), with improvements coinciding with antibiotic treatment (Fig. 1).

All CON bulls remained within a PCV range of 34 – 45% (mean = 40%, median = 41%) for the duration of the study (Fig. 1, Supplemental Table 1). CON bulls maintained normal body temperatures (range: 37.8 − 38.9 °C); however, their temperatures occasionally fluctuated outside of that range when the ambient temperature was < 37.2 °C (Fig. 1, Supplemental Table 2).

**Table 1:** Changes in bull packed cell volume (PCV) during a course of clinical anaplasmosis. Mean percent (%) PCV change in *A. marginale*-infected (ANA) bulls compared to baseline and unchallenged control (CON) bulls over time

						Comp. to Unchallenged	
**Endpoint**	**Phase**	**Day**	**Treatment**	**Mean % Change**	**P-value for**	**Diff. in**	**P-value for**
					**Testing %change≠0**	**Mean % Change**	**Testing Diff.≠0**
PCV	Incubation	3	ANA	2%	0.261	0%	0.985
			CON	2%	0.253	.	.
		8	ANA	1%	0.562	0%	0.969
			CON	1%	0.528	.	.
		10	ANA	4%	0.164	6%	0.154
			CON	-2%	0.48	.	.
		14	ANA	3%	0.29	5%	0.153
			CON	-3%	0.272	.	.
		17	ANA	3%	0.322	6%	0.131
			CON	-4%	0.196	.	.
		21	ANA	-15%	0.065	-9%	0.351
			CON	-6%	0.363	.	.
	Clinical Disease	24	ANA	-42%	0.006	-35%	0.034
			CON	-7%	0.39	.	.
		26	ANA	-54%	0.001	-51%	0.006
			CON	-4%	0.62	.	.
		28	ANA	-64%	< 0.001	-54%	0.002
			CON	-10%	0.133	.	.
		30	ANA	-57%	< 0.001	-54%	< 0.001
			CON	-3%	0.378	.	.
		32	ANA	-49%	< 0.001	-46%	< 0.001
			CON	-3%	0.395	.	.
	Recovery	35	ANA	-38%	< 0.001	-34%	< 0.001
			CON	-4%	0.133	.	.
		38	ANA	-29%	< 0.001	-23%	0.008
			CON	-6%	0.141	.	.
		42	ANA	-19%	< 0.001	-12%	0.003
			CON	-7%	0.007	.	.
	Resolution	49	ANA	-8%	0.018	-3%	0.304
			CON	-4%	0.092	.	.
		56	ANA	-14%	0.096	-10%	0.324
			CON	-4%	0.594	.	.
		63	ANA	-21%	0.019	-17%	0.1
			CON	-4%	0.465	.	.
		70	ANA	-19%	0.022	-17%	0.083
			CON	-2%	0.715	.	.
		77	ANA	-9%	0.059	-1%	0.848
			CON	-8%	0.08	.	.
		84	ANA	0%	0.907	-1%	0.766
			CON	1%	0.596	.	.
		98	ANA	1%	0.811	7%	0.272
			CON	-6%	0.197	.	.
		112	ANA	-2%	0.562	11%	0.102
			CON	-13%	0.022	.	.

### Progression of *A. marginale* infection and bull seroconversion

Bacteremia was first detected via qPCR in all ANA bulls by 3 days post-inoculation. Bacteremia peaked 21 to 26 days post-inoculation. The average bacteremia peak was 8.75E + 08 *A. marginale*/mL blood (median = 8.95E + 08, range: 4.51E + 08 – 1.28E + 09 *A. marginale*/mL blood). All ANA bulls transitioned into the PI disease state with resolution of clinical signs. On the last day of the study, average bacteremia was 2.40E + 07 *A. marginale*/mL blood (median = 2.58E + 07, range: 1.23E + 07 – 3.4E + 07 *A. marginale*/mL). All bulls remained infected with *A. marginale*, despite treatment with oxytetracycline (8553- two treatments; 9431- one treatment; 9528- three treatments). All CON bulls remained qPCR negative for *A. marginale* throughout the study.

Percent parasitized erythrocytes evaluation began 26 days after inoculation (Supplemental Tables 1, Supplemental Fig. 1). The highest PPE detected was 72.3%, 32 days after inoculation (8553). Average peak PPE was 57.6% (median = 51.4%, range: 49.0 − 72.3%). All ANA bulls experienced descending, occasional waves of PPE levels after resolution of clinical signs, never again reaching the peaks observed during the height of clinical disease. On the last day of the study, the average PPE level was 19.3% (median = 20.5%, range: 12.3 − 25.2%). No *A. marginale* parasitized erythrocytes were ever observed in CON bulls at any point in the study.

Earliest seroconversion was detected 10 days post-inoculation (9528). By 17 days post-inoculation, all bulls in the ANA group (8553, 9431) had seroconverted and remained seropositive for the remainder of the study. All CON bulls remained negative for *A. marginale* via PCR and visual examination of stained blood smears (Fig. 1, Supplemental Table 1). CON bulls remained seronegative throughout the study.

### Impact of clinical anaplasmosis on bull breeding soundness examination metrics

#### Overview

Breeding soundness examination results are presented overlaid with different periods of clinical anaplasmosis progression: baseline (time prior to *A. marginale* inoculation), incubation period (period of time prior to display of clinical signs), clinical disease (period of time when clinical signs - fever and anemia - are exhibited), recovery (period of time when clinical signs are returning towards baseline values), and resolution (period of time after anemia and fever are fully resolved with values similar to baseline values).

The occurrence of clinical anaplasmosis coincided with loss of breeding soundness for a variable but extended time period. All ANA bulls fell below satisfactory breeding potential standards beginning 28 days post-inoculation with *A. marginale*, coinciding with peak bacteremia. Bull 9431 never returned to satisfactory breeding potential within the time span of the study (unsatisfactory for more than 72 days), bull 8553 returned to satisfactory breeding potential 72 days after initial failure, and bull 9528 returned to a satisfactory breeding potential 28 days after initial failure. The CON bulls 7532 and 9600 maintained satisfactory breeding soundness throughout the study, while one bull in the CON group (9550) had unsatisfactory BSE results 67 days after study start and never returned to passing. This bull had changes in testicular texture and size consistent with insult related to extreme cold temperatures experienced during the study period. This bull remained negative for *A. marginale* throughout the study.

#### Physical examination

None of the ANA nor the CON bulls developed abnormalities related to feet, legs, or eyes. None of the ANA nor the CON bulls developed any internal genitalia abnormalities at any time point via examination by rectal palpation. All ANA and CON bulls started with and maintained their ability to extend their penis, acquire an erection, and ejaculate via electroejaculation. Although not specifically measured, additional physical response changes were observed in some ANA bulls during clinical disease including prolonged reaction time (time to produce ejaculate) and poor erection response. Two bulls in the ANA group (9431, 9528) developed weak erection response (did not achieve full engorgement, remained slightly flaccid at the time of ejaculation) during peak bacteremia (24 days post-inoculation) but returned to satisfactory response within 21 days of peak bacteremia (45 days post-inoculation).

**Fig. 2:**
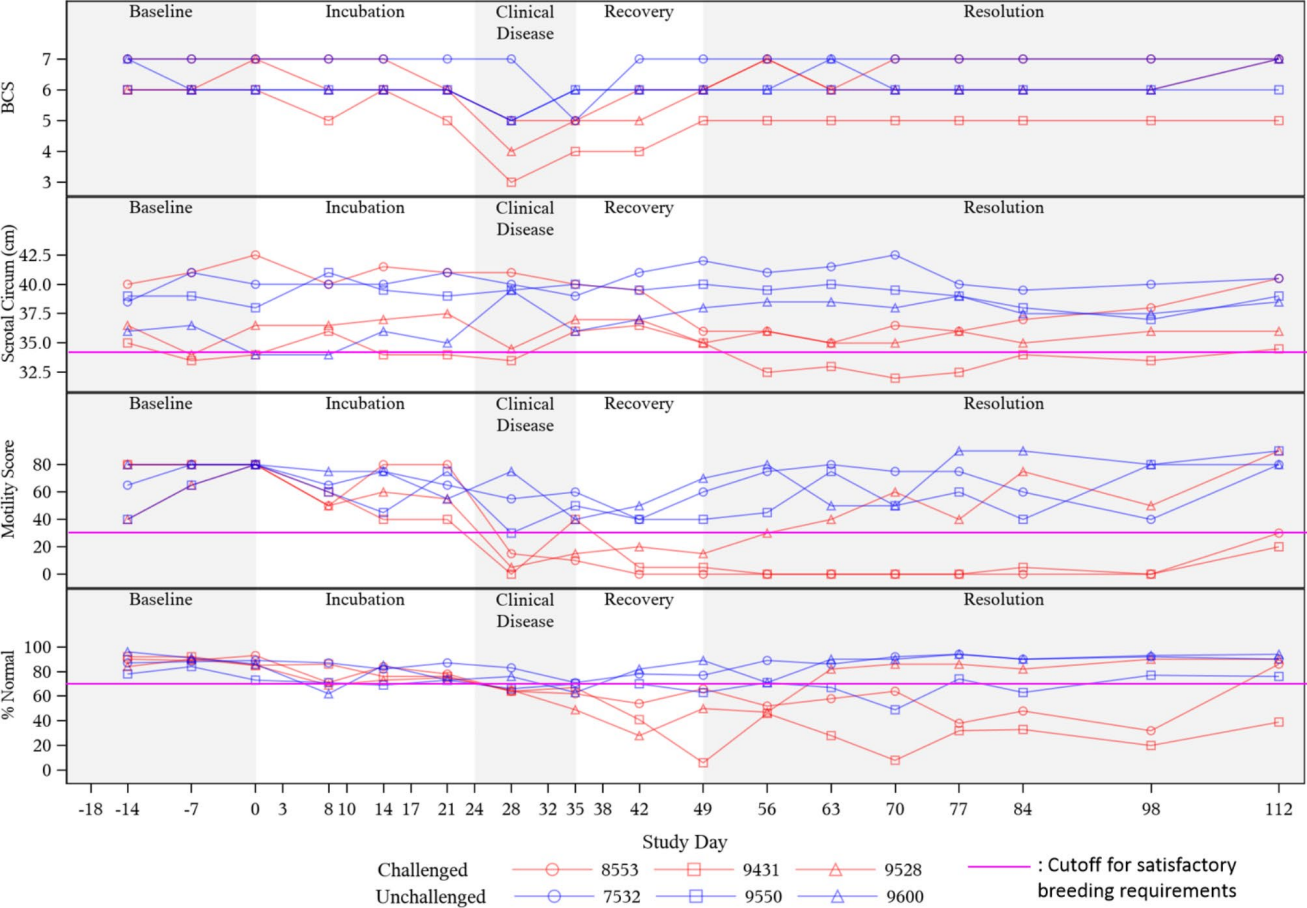
**Changes in bull breeding soundness examination parameters over a course of clinical anaplasmosis.** Top Body Condition Score (BCS) using a 9-point scale [40]. Middle top: Scrotal circumference (cm). Middle bottom: Percentage of progressively motile spermatozoa. Bottom: Percentage of morphologically normal spermatozoa. Figure background shading represents phases of clinical anaplasmosis with Day 0 representing day of *A. marginale* inoculation in challenged bulls.

All ANA bulls had decreased body condition from baseline (Fig. 2, Supplemental Table 3), with transverse processes and ribs becoming more palpable. The most significant decrease in body condition from baseline was by 3 scores in two bulls (9431 and 9528). Notable reductions in body condition score began 21 days post-inoculation. Two bulls (9528 and 8553) returned to baseline body condition 56 days after inoculation; one bull (9431) never returned to baseline but regained some condition before the end of the study. All CON bulls maintained a BSC between 5 and 7 throughout the study.

#### Scrotal circumference and testicular texture

Each ANA bull had decreases in scrotal circumference from baseline (Table 2, Supplemental Table 3). Peak reductions in scrotal circumference were 7% on average in ANA bulls compared to baseline values (Table 2). Greatest percent changes in individual ANA bull scrotal circumference during the study compared to baseline values was 15%, 6.3%, and 4.7% in 8553, 9431, and 9528, respectively. Compared to baseline, average decreases in scrotal circumference in the ANA bulls were not significantly different during clinical disease (Day 28, 2% decrease, *P* = 0.487), but were significant after resolution of clinical disease on Day 70 (7% decrease, *P* = 0.043) (Table 2). Compared to the changes from baseline of time-matched CON bulls, average changes in scrotal circumference were not significant on Day 28 (6% difference, *P* = 0.134), but were significant on Day 70 (12% difference, *P* = 0.02) and neared significance on Day 63 (12% difference, *P* = 0.052). None of the ANA bulls returned to baseline scrotal circumference by the end of the study despite meeting minimum satisfactory standards by the end of the study. One ANA bull (9431) had consistently soft testicles on palpation starting 14 days after inoculation which remained abnormal for approximately 63 days through the end of the study.

Throughout the study among CON bulls, the mean change in scrotal circumference varied 1 – 5% above baseline. Two CON bulls (7532, 9600) had overall scrotal growth from baseline by the end of the study, which was expected as these were younger and still maturing bulls. One CON bull (9550) had an overall 4 cm decrease in scrotal circumference during the study, with consistent decreases most notably in the left testicle starting at Day 77. Despite abnormal testicular palpation, the overall scrotal circumference for this bull met minimum satisfactory standards during the study.

**Table 2:** Changes in scrotal circumference during a course of clinical anaplasmosis. Mean percent (%) scrotal circumference (cm) change in *A. marginale*-infected (ANA) bulls compared to baseline and unchallenged control (CON) bulls over time

					Compared to unchallenged	
**Phase**	**Day**	**Treatment**	**Mean % change**	**P-value for testing change**	**Diff in mean % change**	**P-value for testing** **diff. ≠0**
**Incubation**	8	ANA	2%	0.58	1%	0.829
		CON	1%	0.797	.	.
	14	ANA	1%	0.228	0%	0.985
		CON	1%	0.236	.	.
	21	ANA	1%	0.423	1%	0.796
		CON	1%	0.643	.	.
**Clinical disease**	28	ANA	-2%	0.487	-6%	0.134
		CON	5%	0.133	.	.
**Recovery**	35	ANA	2%	0.378	1%	0.715
		CON	1%	0.685	.	.
	42	ANA	2%	0.403	-1%	0.792
		CON	3%	0.253	.	.
**Resolution**	49	ANA	-4%	0.285	-9%	0.112
		CON	5%	0.176	.	.
	56	ANA	-5%	0.15	-10%	0.084
		CON	5%	0.219	.	.
	63	ANA	-7%	0.097	-12%	0.052
		CON	5%	0.162	.	.
	70	ANA	-7%	0.043	-12%	0.02
		CON	5%	0.076	.	.
	77	ANA	-5%	0.193	-9%	0.138
		CON	4%	0.35	.	.
	84	ANA	-4%	0.196	-5%	0.244
		CON	1%	0.722	.	.
	98	ANA	-3%	0.344	-3%	0.415
		CON	1%	0.841	.	.
	112	ANA	0%	0.96	-4%	0.235
		CON	4%	0.113	.	.

### Sperm morphology

**Fig. 3:**
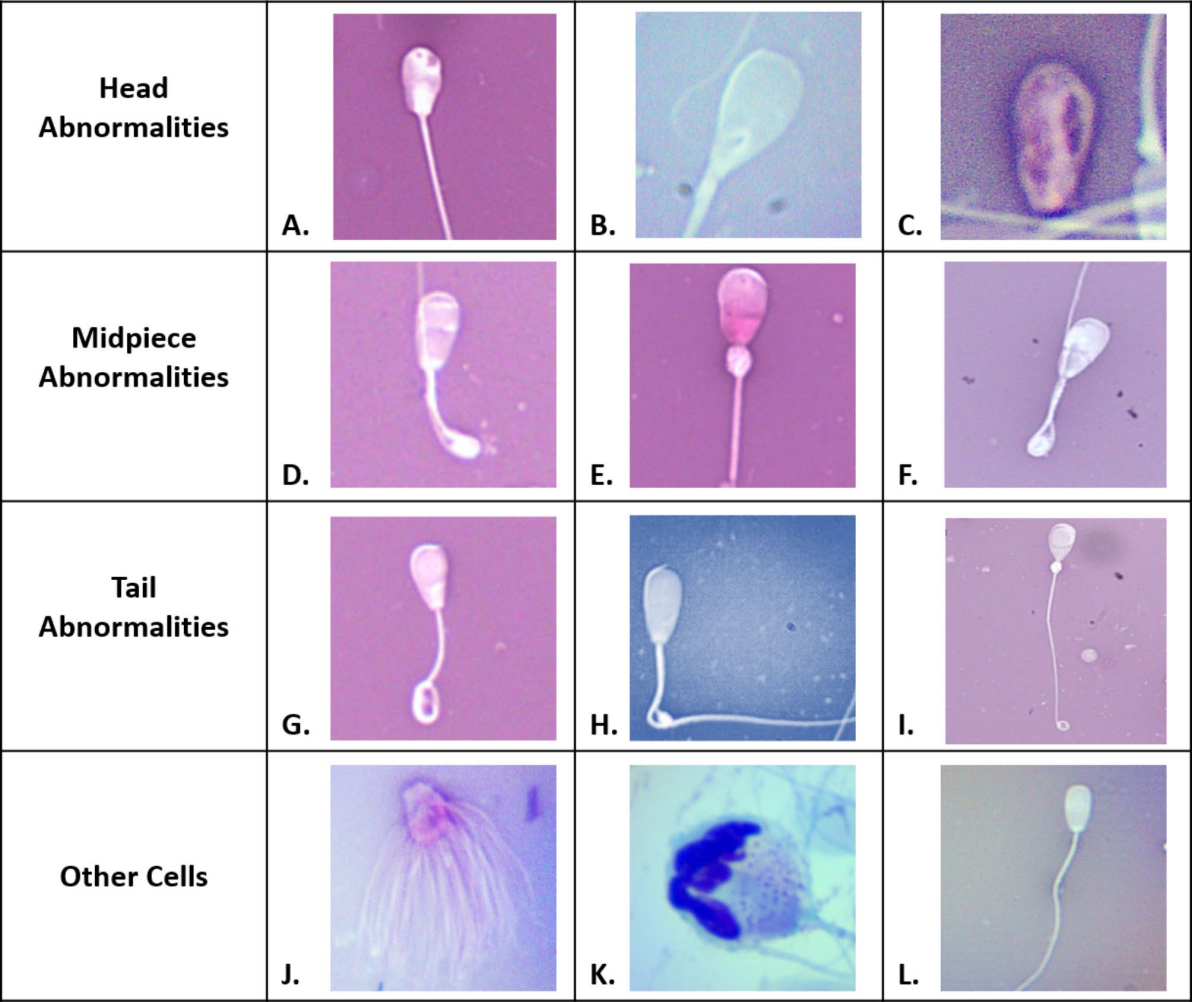
**Abnormal sperm morphologies and other cells in semen samples collected from bulls experimentally-infected with*A. marginale***. Head Abnormalities: (A) nuclear vacuolation; (B) pyriform head; (C) abnormal free head; Midpiece Abnormalities: (D) distal midpiece reflection (DMR); (E) proximal droplet; (F) DMR; Tail Abnormalities: (G) coiled tail; (H) bent principal piece; (I) distal coil with proximal droplet (midpiece); Other Cells: (J) medusa cell; (I) spermatocyte (immature sperm cell); (J) normal, mature sperm cell.

Loss of morphologically normal sperm was observed in all ANA bulls (Fig. 2, Supplemental Table 5). All ANA bulls failed to meet minimum sperm morphology standards beginning at peak clinical anaplasmosis and had a progressively increasing percentage of sperm morphology abnormalities during the anaplasmosis recovery period (Fig. 2). ANA bulls 9528, 8553, and 9431, did not meet minimum sperm morphology requirements for 28-, 70- and at least 84-days, respectively. Bull 9431 never regained satisfactory normal sperm morphology results prior to study completion. The lowest percentage of morphologically normal sperm for bulls 9528, 8553, and 9431 was 28% (42 days post-inoculation), 32% (98 days post-inoculation), and 6% (49 days post-inoculation), respectively, all occurring during clinical anaplasmosis recovery or after resolution. Compared to time-matched CON bulls, the percentage of morphologically normal sperm differed significantly from ANA bulls on Days 28, 42, and 56 (*P* = 0.049, 0.013, 0.026, respectively; Table 3). Morphologically normal sperm (Fig. 3 K) percentages for ANA bulls ranged from 86.3 − 90.7% at baseline; 6 − 32% during peak clinical anaplasmosis; and 39 − 90% after resolution of clinical anaplasmosis. The overall mean percentage of morphologically normal sperm from peak clinical disease through the remainder of the study was 53% for ANA bulls.

**Table 3:** Changes in the percent of progressively motile sperm during a course of clinical anaplasmosis. Mean percent (%) of progressively motile sperm change in *A. marginale*-infected (ANA) bulls compared to baseline and unchallenged control (CON) bulls over time

				Compared to unchallenged	
**Phase**	**Study day**	**Treatment**	**Mean w/ baseline adjusted**	**Diff in mean w/ baseline adjusted**	**P-value for testing** **diff. ≠0**
**Incubation**	8	ANA	53%	-14%	0.053
		CON	67%	.	.
	14	ANA	59%	-6%	0.721
		CON	66%	.	.
	21	ANA	59%	-6%	0.706
		CON	65%	.	.
**Clinical disease**	28	ANA	6%	-49%	0.027
		CON	54%	.	.
**Recovery**	35	ANA	22%	-29%	0.107
		CON	50%	.	.
	42	ANA	9%	-34%	0.018
		CON	43%	.	.
**Resolution**	49	ANA	6%	-51%	0.019
		CON	57%	.	.
	56	ANA	10%	-57%	0.046
		CON	67%	.	.
	63	ANA	15%	-52%	0.015
		CON	67%	.	.
	70	ANA	21%	-36%	0.168
		CON	57%	.	.
	77	ANA	14%	-61%	0.042
		CON	75%	.	.
	84	ANA	28%	-35%	0.344
		CON	62%	.	.
	98	ANA	18%	-47%	0.079
		CON	65%	.	.
	112	ANA	49%	-33%	0.107
		CON	81%	.	.

Collectively, unsatisfactory percentages of sperm head, midpiece, and tail abnormalities were observed in ANA bulls (Fig. 3, Supplemental Table 6, Supplemental Table 7). The most common sperm morphology abnormalities were head abnormalities for bulls 8553 and 9431 and midpiece abnormalities for bull 9528, although head, midpiece, and tail abnormalities were observed in all ANA bulls. Among ANA bulls, the most common types of head abnormalities were nuclear vacuolation, pyriform heads, and free abnormal heads (Fig. 3 A-C). The most common types of midpiece abnormalities were distal midpiece reflections (DMRs), and proximal droplets (Fig. 3D-F). The most common types of tail abnormalities were coiled tails and, to a lesser extent, broken principal pieces (Fig. 3 F-H). Other abnormal cell types observed in the semen of ANA bulls included immature sperm cells (i.e. spermatocytes, medusa cells) (Fig. 3I J). Erythrocytes were never observed in Wright-Giemsa stained semen samples.

For CON bulls 7532 and 9600, the percentage of morphologically normal sperm remained above 70% throughout the study (Fig. 2). One bull in the CON group (9550) had sporadic sperm morphology scores below minimum requirements for passing during the study, occurring on study Day 14, 28, 49, 63, 70, and 84 (Fig. 2). Collectively, morphologically normal sperm percentages for CON bulls ranged from 49-96% throughout the study with an average of 79%

### Sperm motility

**Fig. 4:**
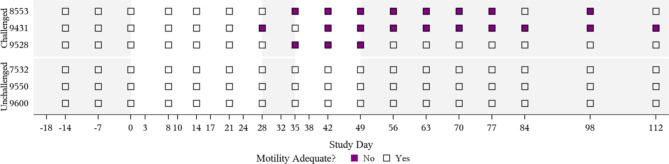
**Maintenance of satisfactory sperm motility scores in bulls experimentally infected with*A. marginale***. Semen samples from bulls experimentally infected with *A. marginale* were assessed for satisfactory (≥ 30% progressively motile sperm) sperm motility compared to time-matched uninfected control bulls. Figure background shading represents disease course of clinical anaplasmosis with Day 0 representing day of *A. inoculation* in challenged bulls.

Loss of progressive sperm motility was observed in all ANA bulls, with sperm motility below minimum satisfactory standards first noted during peak bacteremia and clinical disease (Figs. 2 and 4; Table 4, Supplemental Table 4). ANA bulls 9528 and 8553 fell below the minimum satisfactory sperm motility standards beginning 35 days post-inoculation and consistently remained below these standards for 28- and 70-days, respectively (Fig. 4). ANA bull 9431 fell below the minimum satisfactory sperm motility standards beginning 28 days post-inoculation, rebounded to meeting minimum standards at Day 35, and then consistently fell below minimum satisfactory standards from Day 42 until the end of the study (at least 70 days) (Fig. 4). The percentage of progressively motile sperm for the ANA group bulls ranged from 62 − 80% at baseline; 0 − 5% during clinical anaplasmosis; and 20 − 90% on the last day of the study (Fig. 2). Overall mean percentage of progressively motile sperm from Day 28 (peak clinical disease) to the study end for the ANA group was 17%. Compared to time-matched CON bulls, mean progressive sperm motility was significantly lower in ANA bulls beginning at peak clinical disease on Day 28 (49% difference, *P* = 0.027), and for 35 days after that (Tables 3 and 4).

**Table 4:** Changes in the percent of morphologically normal sperm during a course of clinical anaplasmosis. Mean percent (%) of normal morphology sperm change in *A. marginale*-infected (ANA) bulls compared to baseline and unchallenged control (CON) bulls over time

				Compared to unchallenged	
**Phase**	**Study day**	**Treatment**	**Mean w/ baseline adjusted**	**Diff. in mean w/ baseline adjusted**	**P-value for testing diff ≠ 0**
**Incubation**	8	ANA	75%	2%	0.881
		CON	73%	.	.
	14	ANA	76%	-5%	0.129
		CON	81%	.	.
	21	ANA	76%	-3%	0.682
		CON	78%	.	.
**Clinical disease**	28	ANA	63%	-13%	0.049
		CON	76%	.	.
**Recovery**	35	ANA	59%	-9%	0.318
		CON	68%	.	.
	42	ANA	39%	-40%	0.013
		CON	79%	.	.
**Resolution**	49	ANA	38%	-41%	0.169
		CON	79%	.	.
	56	ANA	48%	-30%	0.026
		CON	78%	.	.
	63	ANA	55%	-27%	0.279
		CON	82%	.	.
	70	ANA	49%	-31%	0.402
		CON	80%	.	.
	77	ANA	52%	-36%	0.21
		CON	88%	.	.
	84	ANA	53%	-30%	0.24
		CON	83%	.	.
	98	ANA	48%	-39%	0.253
		CON	87%	.	.
	112	ANA	70%	-17%	0.466
		CON	88%	.	.

All CON bulls remained above the 30% progressive motility minimum satisfactory standard throughout the study. Progressive sperm motility among CON bulls ranged from 30 to 90%. Overall, the mean percentage of progressively motile sperm for the CON group from Day 28 to study end was 62%, nearly four times the mean of the time-matched ANA group.

## Discussion

In this pilot study, we demonstrate that clinical anaplasmosis negatively impacts beef bull breeding potential in animals previously determined of satisfactory breeding potential. Further, loss of satisfactory breeding potential occurred for an extended time period in bulls newly infected with *A. marginale*, including past resolution of clinical anaplasmosis. All experimentally-infected ANA bulls developed classic clinical signs of acute anaplasmosis, including anemia, fever, and loss of body condition. Upon peak *A. marginale* infection and accompanying clinical disease, bulls exhibited reductions in percentage of morphologically normal sperm, reduced progressive sperm motility, decreased scrotal circumference, and loss of body condition. Loss of satisfactory breeding potential status varied in duration among ANA bulls, but an ultimate return to satisfactory breeding potential was observed in 66.7% (2/3) of the ANA bulls.

Efficient spermatogenesis requires healthy testicular tissue, hormone balance, overall bull health, and appropriate environmental conditions [17, 26, 44]. The process of spermatogenesis from immature cell to the mature sperm cell in the bull typically takes 61 days [44]. Insults to the spermatogenic cycle can affect breeding potential by limiting production of morphologically normal and progressively motile, mature sperm. Location of insult and stage of sperm cell development at the time of insult often determine when these defects are observed in the ejaculate [6]. Environmental and external insults are typically observed more immediately in the ejaculate, while systemic insults may take weeks to be observed in the ejaculate [41]. For example, changes in ambient temperature typically affect sperm in the distal-most aspect of the spermatogenic cycle and morphologic sperm abnormalities associated with this type of insult typically resolve within days as affected sperm are naturally expelled [26]. In contrast, systemic internal insults such as oxidative stress and impaired scrotal thermoregulation from anemia and fever [45–47] can cause direct damage to testicular tissue, requiring a full sperm maturation cycle after resolution of the insult to achieve a successful load if no permanent damage occurs to the testicular parenchyma. Effects from these systemic, internal insults are seen in the ejaculate much later, up to weeks after the insult.

Anaplasmosis causes a hemolytic anemia through removal of infected red blood cells by the reticuloendothelial system [48]. Anemic animals can experience reproductive tissue hypoxia leading to oxidative stress which can interfere with spermatogenesis, hormonal influences, and directly on the mature sperm cell, overall reducing fertility [19–22, 49]. Concurrent with PCV nadirs, our ANA bulls developed significant reductions in progressive sperm motility that continued beyond resolution of anemia (Fig. 2). Although morphologic sperm abnormalities increased to unsatisfactory levels concurrently with PCV nadirs, further reductions in morphologically normal sperm counts were observed for weeks after PCV nadirs (Fig. 2). These findings are consistent with previous studies evaluating BSE-related outcomes in bulls undergoing disease leading to anemia [16, 20, 50, 51]. However, one study assessing the effects of *Theileria orientalis*, another tick-borne pathogen that causes anemia and fever, in Friesian bulls in New Zealand, found no effect on sperm quality parameters despite reductions in libido [52]. Possible explanations for this discrepancy include less severe PCV nadirs (mean PCV nadir 25%) and more mild systemic infection (no fever, no overt clinical signs) in that study compared to what was observed among ANA bulls (mean PCV nadir 15%, fever, overt clinical signs, Supplemental Table 1) in our study.

As with classic clinical anaplasmosis, all ANA bulls developed acute fever (Fig. 1). Increased metabolic rate and oxygen consumption during fever impair sperm cells and their maturation due to hypoxia in the testicular tissue [2, 15, 16, 24, 53]. In their review, Kastelic et al. [25], described several studies demonstrating that scrotal insulation and increased testicular temperature alter normal sperm morphology, motility, and concentration of semen samples beyond the time of insulation. In our study, even when bulls recovered from clinical anaplasmosis and appeared healthy, semen quality continued to be impaired for months after peak infection. A study by Wildeus and Entwistle documented how various morphologic sperm abnormalities occurred at different time points subsequent to the insult in *Bos indicus* x *Bos Taurus* bulls with insulated scrota and elevated testicular temperature [54]. In that study, head abnormalities began 6 days after elevated scrotal temperature and lasted 14 days after insulation. Tail and midpiece abnormalities emerged 12 and 17 days after insult, respectively, and remained present for at least 23 days. In our study, sperm morphology fell below minimum satisfactory standards in the ANA bulls starting at peak clinical disease when they experienced the highest body temperatures. Head, midpiece, and tail abnormalities peaked 42-, 14-, and 21-days post peak fever, respectively (Supplemental Table 6). All ANA bulls had morphologically normal sperm percentages below minimum satisfactory standards for 35 to at least 84 days post-fever. Despite experimental differences (e.g. cause and duration of insult) between the Wildeus study and ours, recovery to satisfactory breeding potential is in agreement with the spermatogenic cycle after resolution of the insult [41].

Most bulls of satisfactory breeding potential will have some proportion of morphologically abnormal sperm, though this value should always be less than 30%. These expected abnormalities can be due to post-ejaculate sample handling or cold shock of the sample. Ejaculate with > 30% morphologically abnormal sperm likely indicates a change in bull health status and a reduced likelihood of reproductive success (e.g. decreased fertilization efficiency, conception rate, embryonic development) [24, 55]. The specific morphologic abnormality can assist in deducing the location and potential cause of the insult [41, 56]. In this study, the primary insult in the ANA bulls was fever and anemia from acute anaplasmosis, that likely affected spermatogenesis at both the cellular and tissue level. Regulation of testicular temperature within the scrotum is incredibly responsive to temperature changes. Anything that interferes with the thermoregulatory capacity within the scrotum— including increases in deep body temperature like fever—can cause damage to spermatogenic tissue and epithelium and lead to morphologically abnormal sperm [57]. The anatomy and physiology of sperm cells make them particularly vulnerable to the effects of oxidative stress caused by anemia and fever, which can further lead to morphologic abnormalities [58, 59]. Collectively, the morphologic sperm abnormalities commonly observed in the ANA bulls during this study reflect damage to both testicular tissue and sperm cells. The presence of specific abnormalities indicate that multiple phases of the spermatogenic cycle were affected, which would be consistent with anaplasmosis causing a systemic disease. The observed abnormalities could reasonably be explained by impaired scrotal thermoregulation and oxidative stress from fever, anemia, and systemic illness caused by acute anaplasmosis. While histopathology is required to provide evidence of direct testicular damage, the presence and persistence of abnormal sperm cells in ANA bulls further suggests that clinical anaplasmosis results in testicular damage. In a production setting, the implication of these morphologic sperm abnormalities points to decreased likelihood of fertilization in the female [41, 46, 55, 60, 61].

Presence of other abnormal cell types were also observed in ANA bull ejaculates. Spermatocytes (Fig. 4 J), or immature sperm cells, were observed in ANA bulls 8553 and 9431 starting 21 days after inoculation prior to clinical disease, peaked at 6 weeks after inoculation, and continued for several weeks after resolution of clinical disease. The presence of high spermatocyte numbers can indicate testicular degeneration, regeneration, or immaturity based on history and other cells present in the spermiogram. In this study, the high number of spermatocytes was likely associated with a regenerative response after transient damage to testicular tissue, with a full spermatogenic cycle required for bulls to achieve mature sperm cells in the ejaculate [62]. Medusa cells (Fig. 4 K) were observed in ANA bulls 8553 and 9431 for 6 consecutive weeks and 1 week, respectively. Medusa cells can be an indicator of significant testicular or epididymal pathology when present in increased numbers [63]. Spermatocytes and medusa cells were not observed for ANA bull 9528; however, teratoids were uniquely observed in this bull for 5 weeks. Although presence of specific abnormal cell types varied in ANA bull ejaculate, the presence of spermatocytes, medusa cells, and/or teratoids collectively suggests impaired spermatogenesis and damage to the seminiferous epithelium, a precursor or indicator of testicular degeneration [24, 26, 62]. No white blood cells were observed in spermiograms of ANA or CON bulls, indicating that loss of breeding soundness in ANA bulls was likely due to systemic disease and less so due to a local inflammatory or infectious process in bull reproductive tissues (e.g. epididymitis, orchitis). Additionally, no red blood cells were observed in semen samples, nor was *A. marginale* organism visually detected in any stained semen preparations. Interestingly, *A. marginale* was inconsistently detected via qPCR in several ANA bull semen samples (semen was tested by qPCR out of curiosity). As *A. marginale* resides in red blood cells, these findings are curious, and may simply be due to sample contamination; however, contamination of semen samples with frank blood was never observed. Further research is required to identify whether PCR-positive semen from infected bulls poses a transmission risk.

Annual breeding soundness examination, including assessment of physical exam parameters, sperm morphology, and progressive sperm motility is one of the most cost-effective measures to confirm satisfactory bull breeding potential [6, 7, 64]. When a bull does not meet any of the minimum parameters for passing a BSE, it’s given an “unsatisfactory” or “deferred” breeding status. The latter is typically given when the BSE changes appear to be transient, especially when performed around extreme weather conditions or on younger animals. Current recommendations for bulls not passing a BSE with a “deferred” status typically include waiting 60 days before re-examination (based on the normal spermatogenic cycle) [41, 56], although sperm-related changes due to ambient temperatures typically resolve in a matter of days [26]. A bull is assigned an “unsatisfactory” breeding status when the BSE changes are associated with more permanent abnormalities, such as changes to testicular size or texture, or when multiple deferred BSEs have already occurred. Two of the ANA bulls in this study (8553, 9431) did not return to a satisfactory breeding status for at least 63 days after resolution of clinical anaplasmosis, longer than the typical 61-day spermatogenesis cycle and recommended re-testing period for unsatisfactory bulls. Importantly, bulls that survive clinical anaplasmosis can return to satisfactory breeding potential (e.g. 8553, 9528), however, they may require a longer period before re-testing, such as bull 8553 that required 72 days to return to satisfactory breeding potential. Bull 9431, that failed to recover satisfactory breeding potential within the study period (72 days past initial failure), may never pass if the testicular tissue was permanently damaged during clinical disease. Our pilot study demonstrates that bulls recovered from clinical anaplasmosis can have extended transient or possibly lasting testicular damage leading to delays in production of normal mature sperm. Future studies incorporating testicular biopsies could address how clinical anaplasmosis damages testicular tissue and to what degree this damage is reversible.

Our findings are similar to an earlier study examining the impact of anaplasmosis on bull breeding soundness by Swift et al. in 1979 [16]. In that study, experimentally-infected bulls developed anemia (mean nadir PCV of 21%) and reproductive changes that included unsatisfactory sperm motility and morphology, testicular degeneration, and reduced libido. Two of the four *A. marginale* inoculated bulls in that study demonstrated improvements in sperm morphology after clinical disease resolved, similar to the outcome for the bulls in our study. Swift et al. further observed reduced libido response to cycling cows by bulls during peak clinical disease. Bull behavior around cycling females was not investigated in our study. Despite a difference of 40 years, differences in equipment and reagents, and likely different *A. marginale* strains, our study and the Swift study had remarkably similar outcomes. Despite a small sample size, our findings agree with those of Swift et al. on how anemia and fever can reduce bull breeding potential.

Building on the results of this pilot study, future studies are needed to mechanistically address how individual hallmark clinical signs of anaplasmosis (e.g. anemia and fever) specifically affect semen quality and reproductive success. Additional variables that could further affect clinical anaplasmosis outcomes and contribute to bull breeding soundness include bull signalment, nutrition status, and co-morbidities. Incorporation of testicular histopathology, ultrasonography, and thermography would enable characterization of anaplasmosis-associated testicular degeneration and injury as prognostication for return to breeding soundness. This is especially important for veterinarians offering recommendations to producers to consider a longer bull retesting window. If the damage caused to the testicle is reversible, retaining bulls longer for retesting is more economic than culling valuable bulls capable of producing viable sperm. However, if testicular damage from disease is irreversible and the bull is retained in the herd for the sake of a longer retesting window, the cost the producer incurs waiting to cull an infertile bull is counted as a loss. Additionally, detection of *A. marginale* DNA in semen samples also warrants effort into whether semen directly, or breeding activities that can cause reproductive mucosal damage, contribute to disease transmission. Finally, whether bulls with chronic anaplasmosis exhibit lower satisfactory semen quality parameters, would be useful to assess as most cattle that survive clinical anaplasmosis develop chronic anaplasmosis.

## Conclusion

Pervasive and expanding infectious diseases of cattle such as bovine anaplasmosis hampers the potential for profitable beef production. Under the conditions of our pilot study, we demonstrate that clinical anaplasmosis causes reduced breeding potential through reduced semen quality, scrotal circumference, and body condition in bulls with previous satisfactory breeding potential. The most striking reductions were in sperm motility and morphology. Although clinical anaplasmosis was associated with reductions in several breeding soundness evaluation metrics, this loss was temporary and a return to satisfactory breeding potential was observed for two of three *A. marginale*-infected bulls. Anaplasmosis should be considered as a differential-diagnosis for bulls with unsatisfactory BSE results or poor reproductive performance in anaplasmosis-endemic areas, especially if there is evidence of recent seroconversion. Veterinarians performing BSEs should consider a longer retesting window of 90 days or longer for deferred bulls, and anaplasmosis testing for breeding bulls in anaplasmosis-endemic areas. Producers should take caution when introducing anaplasmosis-naïve, reproductively sound bulls into an anaplasmosis-endemic herd. The results of our study demonstrate the detrimental effects of clinical anaplasmosis on bull breeding potential, thus it is imperative that cow-calf herd management, testing, and treatment protocols for *A. marginale* consider risks and impacts of the disease in both females and bulls.

## Electronic supplementary material

Below is the link to the electronic supplementary material.

Supplementary Material 1

Supplementary Material 2

Supplementary Material 3

Supplementary Material 4

Supplementary Material 5

Supplementary Material 6

Supplementary Material 7

Supplementary Material 8

## Data Availability

The datasets used and/or analyzed during the current study are included in the published article or supplementary files. Sequences from this study identifying the *A. marginale* challenge strain Msp1a genotype were submitted to GenBank and are available at the following accession numbers: ON597854-ON97859.

## List of abbreviations

ANA: *A. marginale*-infected group.
BSE: Breeding Soundness Examination.
BSEs: Breeding Soundness Examinations.
cELISA: Competitive Enzyme-linked immunosorbent assay.
CON: Uninfected control group.
DMR: Distal Midpiece Reflection.
EDTA: Ethylenediaminetetraacetic acid.
PBS: Phosphate buffered saline.
PCV: Packed cell volume.
PI: Persistently infected.
PPE: Percent parasitized erythrocytes.
qPCR: Quantitative Polymerase Chain Reaction.
SFT: Society for Theriogenology.
