# Supplementary material for: Satisfactory breeding potential is transiently eliminated in beef bulls with clinical anaplasmosis
Source: BMC Vet Res. 2022 Oct 29;18:381. doi: 10.1186/s12917-022-03470-7 (PMC9617051; doi:10.1186/s12917-022-03470-7)
Supplement: Supplementary file 1 — Supplementary Material 1 [file 12917_2022_3470_MOESM1_ESM.docx]

Supplemental Tables

**Supplemental Table 1**. Summary statistics for blood parameters associated with *A. marginale* infection. Mean, minimum and maximum packed cell volume (PCV), bacteremia (*A. marginale* (*Am*)/mL blood), and percent parasitized erythrocytes (PPE) among *A. marginale*-challenged and unchallenged bulls.

|  | | **PCV** | | | | | | **Am/ml blood** | | | | | | **PPE** | | | | | |
| --- | --- | --- | --- | --- | --- | --- | --- | --- | --- | --- | --- | --- | --- | --- | --- | --- | --- | --- | --- |
|  |  | **ANA** | | | **CON** | | | **ANA** | | | **CON** | | | **ANA** | | | **CON** | | |
|  |  | **Mean** | **Min** | **Max** | **Mean** | **Min** | **Max** | **Mean** | **Min** | **Max** | **Mean** | **Min** | **Max** | **Mean** | **Min** | **Max** | **Mean** | **Min** | **Max** |
| **Phase** | **Study Day** | 41% | 38% | 45% | 43% | 40% | 44% | 0 | 0 | 0 | 0 | 0 | 0 | . | . | . | . | . | . |
| **Baseline** | **-18** |  |  |  |  |  |  |  |  |  |  |  |  |  |  |  |  |  |  |
|  | **-14** | 42% | 39% | 44% | 40% | 39% | 42% | 0 | 0 | 0 | 0 | 0 | 0 | . | . | . | . | . | . |
|  | **-7** | 41% | 40% | 43% | 42% | 40% | 44% | 0 | 0 | 0 | 0 | 0 | 0 | . | . | . | . | . | . |
|  | **0** | 41% | 39% | 42% | 42% | 41% | 44% | 0 | 0 | 0 | 0 | 0 | 0 | . | . | . | . | . | . |
| **Incubation** | **3** | 42% | 41% | 43% | 43% | 40% | 44% | 7901 | 2273.3 | 11065 | 0 | 0 | 0 | . | . | . | . | . | . |
|  | **8** | 42% | 41% | 42% | 42% | 41% | 44% | 149352 | 50486 | 240157 | 0 | 0 | 0 | . | . | . | . | . | . |
|  | **10** | 43% | 41% | 44% | 41% | 39% | 44% | 1.07E6 | 209331 | 1.56E6 | 0 | 0 | 0 | . | . | . | . | . | . |
|  | **14** | 42% | 39% | 44% | 41% | 40% | 41% | 1.28E7 | 2.57E6 | 2.02E7 | 0 | 0 | 0 | . | . | . | . | . | . |
|  | **17** | 42% | 39% | 45% | 40% | 39% | 42% | 7.18E7 | 7.03E6 | 1.05E8 | 0 | 0 | 0 | . | . | . | . | . | . |
|  | **21** | 35% | 32% | 39% | 39% | 37% | 43% | 3.9E8 | 4.94E7 | 6.68E8 | 0 | 0 | 0 | . | . | . | . | . | . |
| **Clinical Disease** | **24** | 24% | 18% | 30% | 39% | 36% | 41% | 4.97E8 | 2.87E8 | 8.95E8 | 0 | 0 | 0 | . | . | . | . | . | . |
|  | **26** | 19% | 14% | 25% | 40% | 37% | 43% | 8.19E8 | 3.44E8 | 1.28E9 | 0 | 0 | 0 | 33% | 24% | 41% | 0% | 0% | 0% |
|  | **28** | 15% | 13% | 18% | 38% | 34% | 41% | 2.81E8 | 5.48E7 | 4.3E8 | 0 | 0 | 0 | 41% | 34% | 45% | 0% | 0% | 0% |
|  | **30** | 18% | 15% | 19% | 41% | 39% | 42% | 1.44E8 | 2.22E7 | 3.53E8 | 0 | 0 | 0 | 51% | 35% | 72% | 0% | 0% | 0% |
|  | **32** | 21% | 19% | 23% | 41% | 37% | 44% | 7.3E7 | 1.79E7 | 1.71E8 | 0 | 0 | 0 | 58% | 49% | 72% | 0% | 0% | 0% |
| **Recovery** | **35** | 26% | 23% | 29% | 40% | 39% | 41% | 7.28E6 | 5E6 | 1E7 | 0 | 0 | 0 | 50% | 43% | 55% | 0% | 0% | 0% |
|  | **38** | 29% | 27% | 32% | 39% | 37% | 43% | 1.86E6 | 529142 | 2.71E6 | 0 | 0 | 0 | 47% | 45% | 48% | 0% | 0% | 0% |
|  | **42** | 33% | 31% | 35% | 39% | 36% | 41% | 1.13E6 | 91686 | 2.82E6 | 0 | 0 | 0 | 39% | 36% | 45% | 0% | 0% | 0% |
| **Resolution** | **49** | 38% | 37% | 40% | 40% | 37% | 42% | 1.64E7 | 976665 | 4.68E7 | 0 | 0 | 0 | 15% | 8% | 20% | 0% | 0% | 0% |
|  | **56** | 36% | 28% | 42% | 40% | 36% | 45% | 8.58E7 | 4.77E7 | 1.6E8 | 0 | 0 | 0 | 10% | 8% | 12% | 0% | 0% | 0% |
|  | **63** | 33% | 27% | 38% | 40% | 37% | 45% | 1.83E8 | 5.8E7 | 3.4E8 | 0 | 0 | 0 | 15% | 13% | 18% | 0% | 0% | 0% |
|  | **70** | 33% | 29% | 39% | 41% | 38% | 44% | 6.94E7 | 5.46E6 | 1.16E8 | 0 | 0 | 0 | 29% | 14% | 54% | 0% | 0% | 0% |
|  | **77** | 37% | 36% | 39% | 38% | 36% | 43% | 1.36E7 | 7.33E6 | 2.11E7 | 0 | 0 | 0 | 26% | 16% | 38% | 0% | 0% | 0% |
|  | **84** | 41% | 40% | 42% | 42% | 41% | 45% | 3.16E7 | 2.99E6 | 8.53E7 | 0 | 0 | 0 | 14% | 9% | 20% | 0% | 0% | 0% |
|  | **98** | 42% | 39% | 43% | 39% | 36% | 44% | 4.67E6 | 823691 | 7.58E6 | 0 | 0 | 0 | 18% | 13% | 27% | 0% | 0% | 0% |
|  | **112** | 40% | 37% | 43% | 36% | 34% | 41% | 2.4E7 | 1.23E7 | 3.4E7 | 0 | 0 | 0 | 19% | 12% | 25% | 0% | 0% | 0% |

**Supplemental Table 2**: Summary statistics for bull rectal temperatures. Mean, minimum and maximum body temperatures (°C) among *A. marginale*-challenged and unchallenged bulls. Ambient outdoor temperature (°C) is also included.

|  | | **Ambient Temp. (°F)** | **Rectal Temp (°F)** | | | | | |
| --- | --- | --- | --- | --- | --- | --- | --- | --- |
|  |  |  | **ANA** | | | **CON** | | |
|  |  |  | **Mean** | **Min** | **Max** | **Mean** | **Min** | **Max** |
| **Phase** | **Study Day** | 35.0 | 103.1 | 102.3 | 103.7 | 103.9 | 102.3 | 105.7 |
| **Baseline** | **-18** |  |  |  |  |  |  |  |
|  | **-14** | 25.0 | 101.1 | 100.8 | 101.5 | 101.9 | 100.9 | 102.6 |
|  | **-7** | 39.0 | 101.6 | 101.1 | 102.1 | 100.9 | 100.3 | 101.6 |
|  | **0** | 9.0 | 101.4 | 100.5 | 102.0 | 99.2 | 98.4 | 100.3 |
| **Incubation** | **3** | 0.0 | 99.5 | 98.3 | 100.3 | 99.6 | 98.9 | 100.2 |
|  | **8** | 3.0 | 101.6 | 100.0 | 102.6 | 99.5 | 98.9 | 100.0 |
|  | **10** | 18.0 | 101.3 | 100.6 | 101.9 | 100.7 | 100.5 | 100.9 |
|  | **14** | 32.0 | 102.4 | 101.8 | 103.4 | 101.6 | 101.1 | 102.0 |
|  | **17** | 26.0 | 101.8 | 101.2 | 102.2 | 100.9 | 100.3 | 101.8 |
|  | **21** | 39.0 | 102.8 | 101.9 | 104.2 | 100.5 | 100.2 | 100.9 |
| **Clinical Disease** | **24** | 32.0 | 104.2 | 103.6 | 105.1 | 100.8 | 100.3 | 101.4 |
|  | **26** | 46.0 | 103.2 | 101.5 | 104.3 | 100.3 | 100.1 | 100.6 |
|  | **28** | 59.0 | 104.1 | 102.1 | 105.5 | 101.4 | 101.1 | 101.6 |
|  | **30** | 30.0 | 102.2 | 101.9 | 102.5 | 100.9 | 100.1 | 101.3 |
|  | **32** | 43.0 | 102.1 | 101.1 | 102.6 | 101.1 | 100.4 | 102.1 |
| **Recovery** | **35** | 45.0 | 101.7 | 101.2 | 102.0 | 101.0 | 100.7 | 101.2 |
|  | **38** | 37.0 | 101.6 | 101.2 | 102.2 | 100.4 | 99.6 | 100.9 |
|  | **42** | 50.0 | 102.3 | 101.6 | 102.8 | 102.1 | 101.6 | 103.0 |
| **Resolution** | **49** | 46.0 | 100.7 | 100.7 | 100.8 | 100.7 | 100.3 | 101.5 |
|  | **56** | 60.0 | 101.4 | 101.0 | 101.9 | 101.5 | 101.3 | 101.9 |
|  | **63** | 46.0 | 101.6 | 100.7 | 102.2 | 100.7 | 100.2 | 101.4 |
|  | **70** | 34.0 | 100.6 | 100.3 | 100.8 | 99.8 | 99.7 | 99.8 |
|  | **77** | 70.0 | 101.4 | 100.9 | 101.6 | 100.9 | 100.5 | 101.4 |
|  | **84** | 52.0 | 101.4 | 101.1 | 101.8 | 101.0 | 100.7 | 101.2 |
|  | **98** | 72.0 | 101.7 | 101.5 | 102.2 | 101.7 | 101.1 | 102.5 |
|  | **112** | 64.0 | 102.0 | 101.6 | 102.7 | 101.9 | 101.2 | 103.0 |

**Supplemental Table 3**. Summary statistics for bull body condition scores (BCS) and scrotal circumference throughout a course of clinical anaplasmosis. Mean, minimum and maximum BCS and scrotal circumference (cm) among *A. marginale*-challenged and unchallenged bulls. ^1^BSC determined using a 9-point scale (42)

| **Phase** | **Study Day** | **BCS^1^** | | | | | | **Scrotal circumference (cm)** | | | | | |
| --- | --- | --- | --- | --- | --- | --- | --- | --- | --- | --- | --- | --- | --- |
|  |  | **ANA** | | | **CON** | | | **ANA** | | | **CON** | | |
|  |  | **Mean** | **Min** | **Max** | **Mean** | **Min** | **Max** | **Mean** | **Min** | **Max** | **Mean** | **Min** | **Max** |
| **Baseline** | **-14** | 6.3 | 6 | 7 | 6.7 | 6 | 7 | 37.2 | 35.0 | 40.0 | 37.8 | 36.0 | 39.0 |
|  | **-7** | 6.3 | 6 | 7 | 6.3 | 6 | 7 | 36.2 | 33.5 | 41.0 | 38.8 | 36.5 | 41.0 |
|  | **0** | 6.7 | 6 | 7 | 6.3 | 6 | 7 | 37.7 | 34.0 | 42.5 | 37.3 | 34.0 | 40.0 |
| **Incubation** | **8** | 6.0 | 5 | 7 | 6.3 | 6 | 7 | 37.5 | 36.0 | 40.0 | 38.3 | 34.0 | 41.0 |
|  | **14** | 6.3 | 6 | 7 | 6.3 | 6 | 7 | 37.5 | 34.0 | 41.5 | 38.5 | 36.0 | 40.0 |
|  | **21** | 5.7 | 5 | 6 | 6.3 | 6 | 7 | 37.5 | 34.0 | 41.0 | 38.3 | 35.0 | 41.0 |
| **Clinical disease** | **28** | 4.0 | 3 | 5 | 5.7 | 5 | 7 | 36.3 | 33.5 | 41.0 | 39.7 | 39.5 | 40.0 |
| **Recovery** | **35** | 4.7 | 4 | 5 | 5.7 | 5 | 6 | 37.7 | 36.0 | 40.0 | 38.3 | 36.0 | 40.0 |
|  | **42** | 5.0 | 4 | 6 | 6.3 | 6 | 7 | 37.7 | 36.5 | 39.5 | 39.2 | 37.0 | 41.0 |
| **Resolution** | **49** | 5.7 | 5 | 6 | 6.3 | 6 | 7 | 35.3 | 35.0 | 36.0 | 40.0 | 38.0 | 42.0 |
|  | **56** | 6.3 | 5 | 7 | 6.3 | 6 | 7 | 34.8 | 32.5 | 36.0 | 39.7 | 38.5 | 41.0 |
|  | **63** | 5.7 | 5 | 6 | 6.7 | 6 | 7 | 34.3 | 33.0 | 35.0 | 40.0 | 38.5 | 41.5 |
|  | **70** | 6.0 | 5 | 7 | 6.3 | 6 | 7 | 34.5 | 32.0 | 36.5 | 40.0 | 38.0 | 42.5 |
|  | **77** | 6.0 | 5 | 7 | 6.3 | 6 | 7 | 34.8 | 32.5 | 36.0 | 39.3 | 39.0 | 40.0 |
|  | **84** | 6.0 | 5 | 7 | 6.3 | 6 | 7 | 35.3 | 34.0 | 37.0 | 38.3 | 37.5 | 39.5 |
|  | **98** | 6.0 | 5 | 7 | 6.3 | 6 | 7 | 35.8 | 33.5 | 38.0 | 38.2 | 37.0 | 40.0 |
|  | **112** | 6.3 | 5 | 7 | 6.7 | 6 | 7 | 37.0 | 34.5 | 40.5 | 39.3 | 38.5 | 40.5 |

**Supplemental Table 4**. Sperm progressive motility results. Number of *A. marginale*-challenged and unchallenged bulls with a satisfactory percentage (>30%) of sperm with progressive motility; and summary statistics (mean, minimum, maximum) of sperm with progressive motility.

| **Phase** | **Study Day** | **# of bulls with satisfactory progressive sperm motility** | | | **% of sperm with progressive motility** | | | | | |
| --- | --- | --- | --- | --- | --- | --- | --- | --- | --- | --- |
|  |  | **ANA** | | **CON** | **ANA** | | | **CON** | | |
|  |  | **Yes** | **No** | **Yes** | **Mean** | **Min** | **Max** | **Mean** | **Min** | **Max** |
| **Baseline** | **-14** | 3 | . | 3 | 67% | 40% | 80% | 62% | 40% | 80% |
|  | **-7** | 3 | . | 3 | 75% | 65% | 80% | 75% | 65% | 80% |
|  | **0** | 3 | . | 3 | 80% | 80% | 80% | 80% | 80% | 80% |
| **Incubation** | **8** | 3 | . | 3 | 53% | 50% | 60% | 67% | 60% | 75% |
|  | **14** | 3 | . | 3 | 60% | 40% | 80% | 65% | 45% | 75% |
|  | **21** | 3 | . | 3 | 58% | 40% | 80% | 65% | 55% | 75% |
| **Clinical disease** | **28** | 2 | 1 | 3 | 7% | 0% | 15% | 53% | 30% | 75% |
| **Recovery** | **35** | 1 | 2 | 3 | 22% | 10% | 40% | 50% | 40% | 60% |
|  | **42** | . | 3 | 3 | 8% | 0% | 20% | 43% | 40% | 50% |
| **Resolution** | **49** | . | 3 | 3 | 7% | 0% | 15% | 57% | 40% | 70% |
|  | **56** | 1 | 2 | 3 | 10% | 0% | 30% | 67% | 45% | 80% |
|  | **63** | 1 | 2 | 3 | 13% | 0% | 40% | 68% | 50% | 80% |
|  | **70** | 1 | 2 | 3 | 20% | 0% | 60% | 58% | 50% | 75% |
|  | **77** | 2 | 1 | 3 | 13% | 0% | 40% | 75% | 60% | 90% |
|  | **84** | 2 | 1 | 3 | 27% | 0% | 75% | 63% | 40% | 90% |
|  | **98** | 1 | 2 | 3 | 17% | 0% | 50% | 67% | 40% | 80% |
|  | **112** | 2 | 1 | 3 | 47% | 20% | 90% | 83% | 80% | 90% |

**Supplemental Table 5**. Summary statistics for normal morphology sperm. Mean, minimum, and maximum percent of sperm with normal morphology among *A.* *marginale*-challenged and unchallenged bulls.

| **Phase** | **Study Day** | **% of sperm with normal morphology** | | | | | |
| --- | --- | --- | --- | --- | --- | --- | --- |
|  |  | **ANA** | | | **CON** | | |
|  |  | **Mean** | **Min** | **Max** | **Mean** | **Min** | **Max** |
| **Baseline** | **-14** | 89% | 84% | 92% | 87% | 78% | 96% |
|  | **-7** | 90% | 89% | 92% | 88% | 84% | 91% |
|  | **0** | 88% | 85% | 93% | 83% | 73% | 89% |
| **Incubation** | **8** | 75% | 69% | 86% | 73% | 62% | 87% |
|  | **14** | 78% | 73% | 84% | 79% | 69% | 85% |
|  | **21** | 76% | 75% | 78% | 78% | 73% | 87% |
| **Clinical disease** | **28** | 64% | 64% | 65% | 75% | 66% | 83% |
| **Recovery** | **35** | 59% | 49% | 67% | 68% | 63% | 71% |
|  | **42** | 41% | 28% | 54% | 77% | 70% | 82% |
| **Resolution** | **49** | 41% | 6% | 66% | 76% | 63% | 89% |
|  | **56** | 48% | 46% | 52% | 77% | 71% | 89% |
|  | **63** | 56% | 28% | 82% | 81% | 67% | 90% |
|  | **70** | 53% | 8% | 86% | 77% | 49% | 92% |
|  | **77** | 52% | 32% | 86% | 87% | 74% | 94% |
|  | **84** | 54% | 33% | 82% | 81% | 63% | 90% |
|  | **98** | 47% | 20% | 90% | 87% | 77% | 93% |
|  | **112** | 72% | 39% | 90% | 87% | 76% | 94% |

**Supplemental Table 6**. Summary statistics for observed abnormal sperm morphology categories. Mean, minimum, and maximum of abnormal morphology sperm categories among *A.* *marginale*-challenged and unchallenged bulls.

| **Phase** | **Study Day** | **% head abnormalities** | | | | | | **% midpiece abnormalities** | | | | | | **% tail abnormalities** | | | | | |
| --- | --- | --- | --- | --- | --- | --- | --- | --- | --- | --- | --- | --- | --- | --- | --- | --- | --- | --- | --- |
|  |  | **ANA** | | | **CON** | | | **ANA** | | | **CON** | | | **ANA** | | | **CON** | | |
|  |  | **Mean** | **Min** | **Max** | **Mean** | **Min** | **Max** | **Mean** | **Min** | **Max** | **Mean** | **Min** | **Max** | **Mean** | **Min** | **Max** | **Mean** | **Min** | **Max** |
| **Baseline** | **-14** | 5% | 3% | 6% | 7% | 1% | 15% | 4% | 2% | 5% | 3% | 1% | 5% | 3% | 1% | 6% | 3% | 2% | 5% |
|  | **-7** | 3% | 0% | 5% | 8% | 7% | 10% | 2% | 1% | 3% | 2% | 0% | 4% | 5% | 3% | 8% | 2% | 0% | 3% |
|  | **0** | 7% | 1% | 10% | 6% | 2% | 11% | 3% | 1% | 5% | 5% | 2% | 9% | 3% | 1% | 4% | 6% | 5% | 7% |
| **Incubation** | **8** | 3% | 1% | 7% | 3% | 3% | 4% | 14% | 2% | 23% | 17% | 5% | 24% | 7% | 5% | 12% | 6% | 4% | 10% |
|  | **14** | 8% | 5% | 12% | 10% | 3% | 18% | 11% | 10% | 12% | 8% | 6% | 9% | 3% | 1% | 8% | 4% | 3% | 4% |
|  | **21** | 7% | 4% | 10% | 12% | 8% | 17% | 11% | 9% | 12% | 7% | 5% | 9% | 6% | 5% | 7% | 3% | 0% | 8% |
| **Clinical disease** | **28** | 15% | 7% | 23% | 13% | 4% | 24% | 16% | 9% | 25% | 10% | 9% | 10% | 5% | 4% | 7% | 2% | 0% | 4% |
| **Recovery** | **35** | 14% | 4% | 21% | 20% | 8% | 28% | 20% | 10% | 40% | 9% | 3% | 14% | 6% | 4% | 7% | 4% | 1% | 7% |
|  | **42** | 22% | 12% | 32% | 12% | 3% | 25% | 24% | 12% | 38% | 9% | 5% | 12% | 13% | 4% | 22% | 2% | 0% | 3% |
| **Resolution** | **49** | 35% | 16% | 52% | 10% | 1% | 24% | 10% | 6% | 14% | 10% | 7% | 15% | 15% | 4% | 36% | 4% | 3% | 4% |
|  | **56** | 33% | 14% | 44% | 14% | 7% | 20% | 13% | 6% | 26% | 7% | 4% | 12% | 6% | 0% | 13% | 2% | 0% | 3% |
|  | **63** | 31% | 8% | 66% | 13% | 5% | 26% | 6% | 0% | 14% | 2% | 0% | 4% | 7% | 6% | 8% | 4% | 2% | 6% |
|  | **70** | 39% | 8% | 84% | 16% | 4% | 40% | 6% | 3% | 10% | 5% | 2% | 9% | 2% | 2% | 3% | 1% | 1% | 2% |
|  | **77** | 32% | 7% | 54% | 10% | 4% | 22% | 9% | 3% | 15% | 2% | 1% | 4% | 6% | 4% | 11% | 0% | 0% | 1% |
|  | **84** | 25% | 5% | 36% | 11% | 3% | 24% | 15% | 6% | 23% | 4% | 2% | 9% | 6% | 0% | 10% | 4% | 2% | 5% |
|  | **98** | 33% | 4% | 50% | 9% | 3% | 19% | 13% | 2% | 20% | 3% | 3% | 3% | 7% | 4% | 10% | 1% | 0% | 2% |
|  | **112** | 13% | 2% | 33% | 9% | 3% | 19% | 10% | 4% | 20% | 3% | 2% | 5% | 5% | 3% | 8% | 1% | 0% | 1% |
