# Supplementary figures and images for: Satisfactory breeding potential is transiently eliminated in beef bulls with clinical anaplasmosis
Source: BMC Vet Res. 2022 Oct 29;18:381. doi: 10.1186/s12917-022-03470-7 (PMC9617051; doi:10.1186/s12917-022-03470-7)

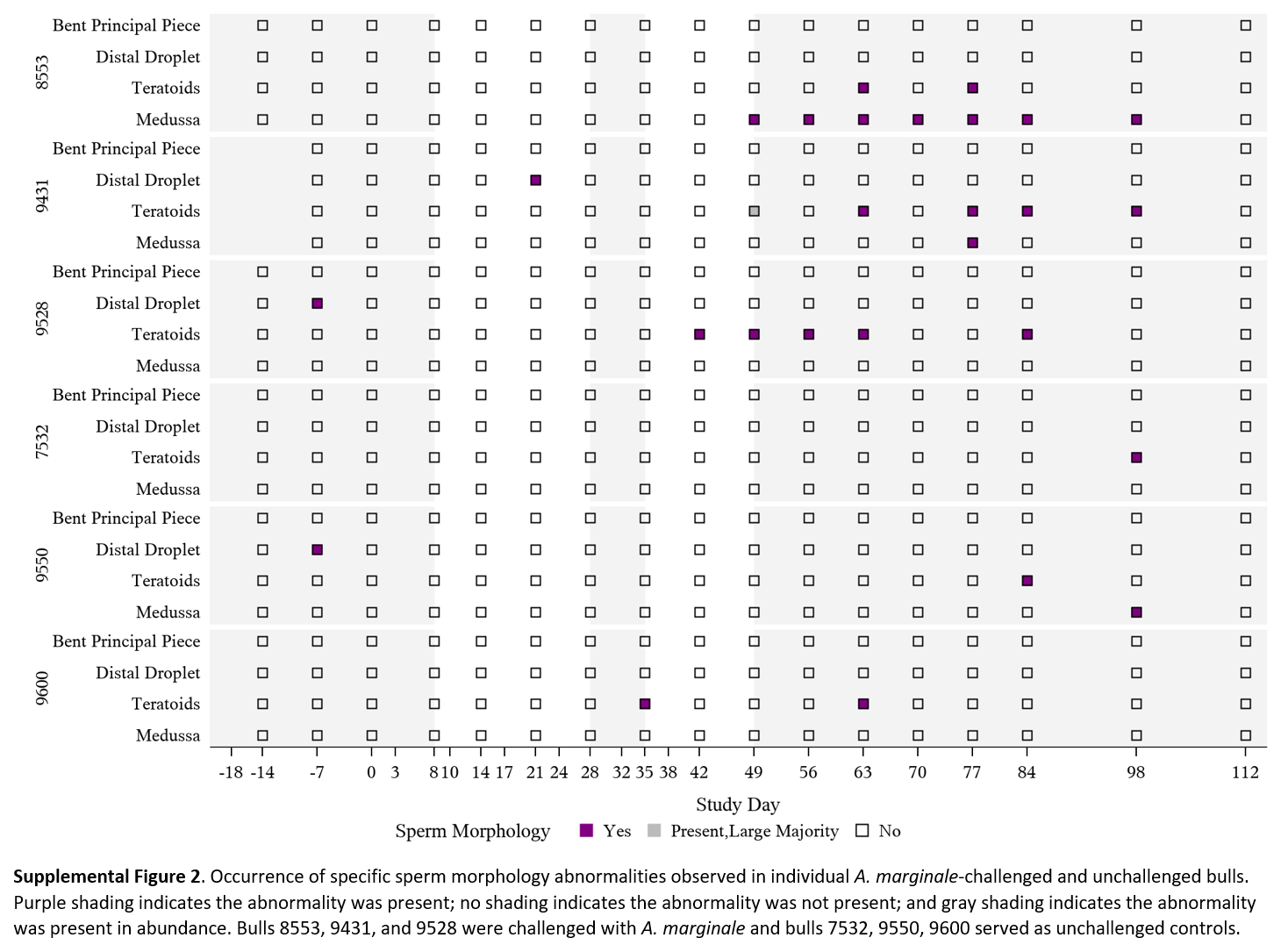

Supplement: Supplementary file 2 — Supplementary Material 2 [file 12917_2022_3470_MOESM2_ESM.png]

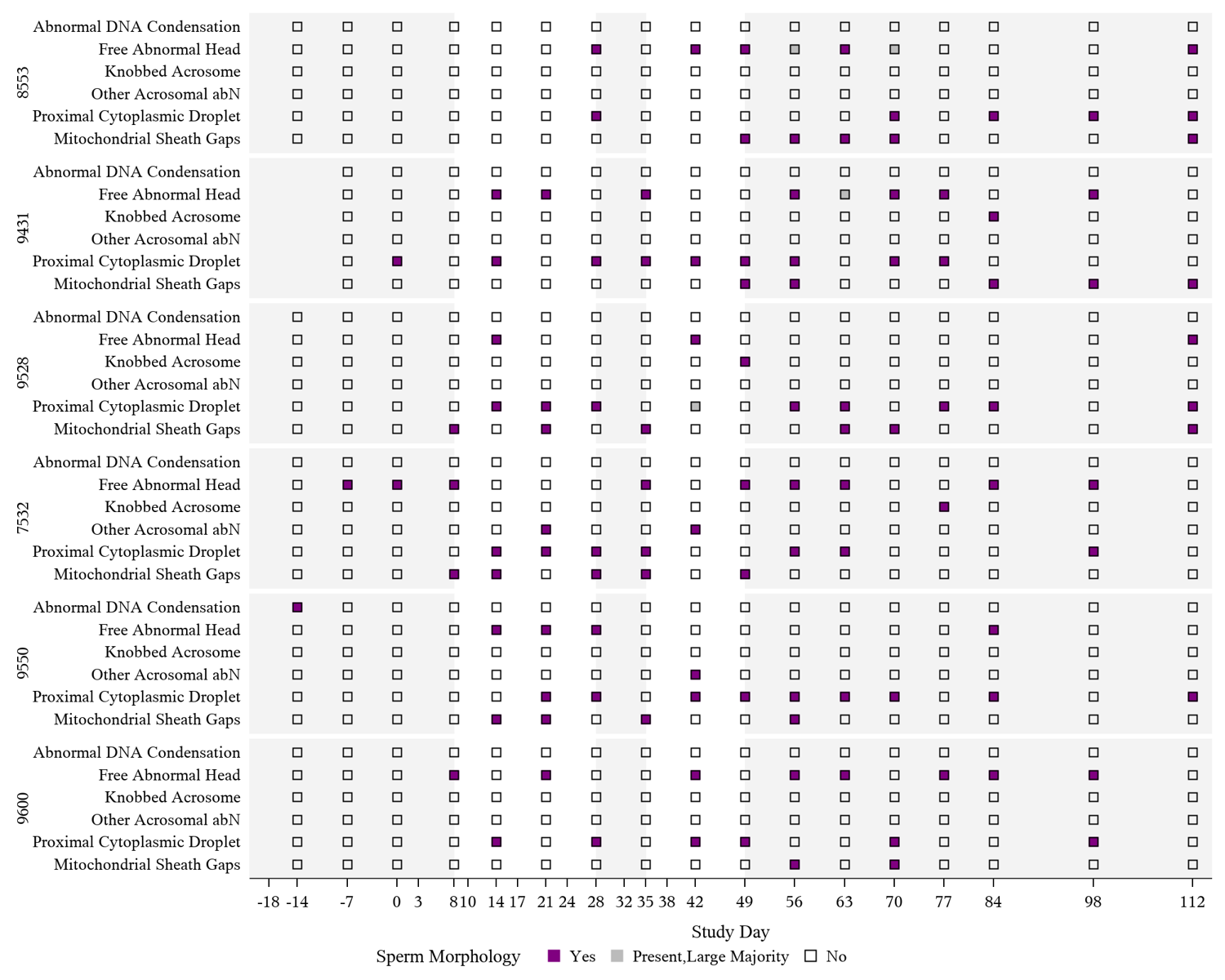

Supplement: Supplementary file 3 — Supplementary Material 3 [file 12917_2022_3470_MOESM3_ESM.png]

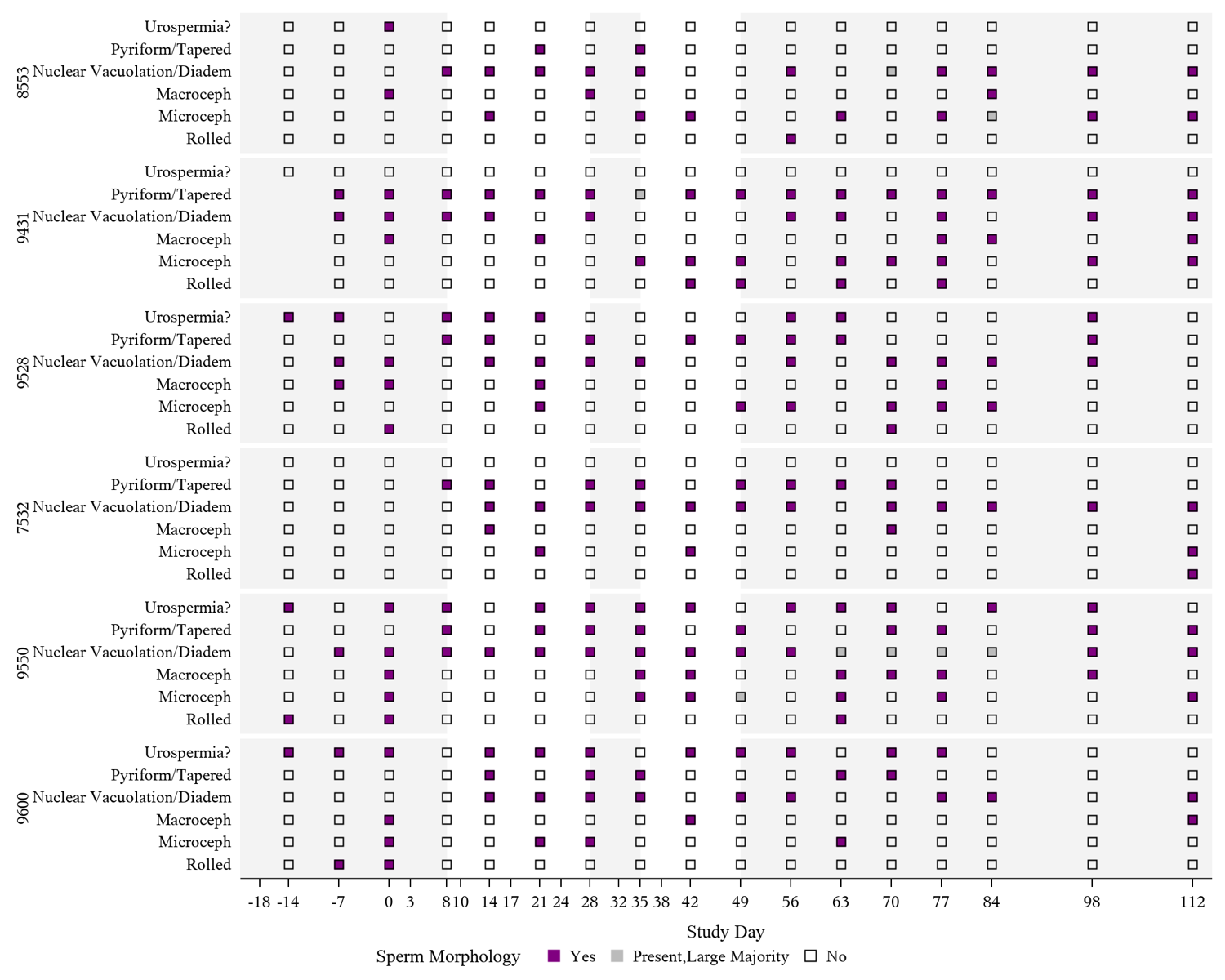

Supplement: Supplementary file 5 — Supplementary Material 5 [file 12917_2022_3470_MOESM5_ESM.png]

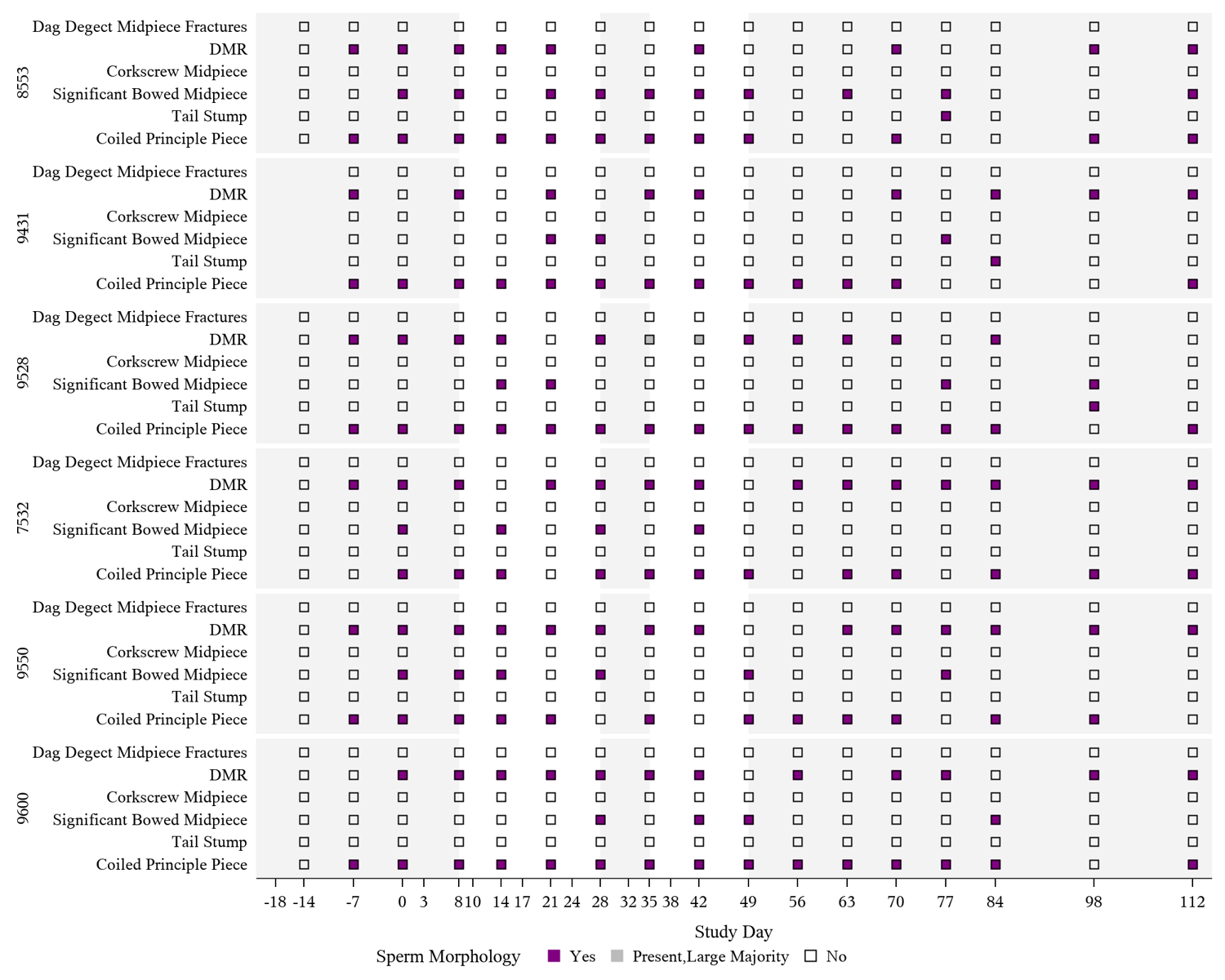

Supplement: Supplementary file 6 — Supplementary Material 6 [file 12917_2022_3470_MOESM6_ESM.png]

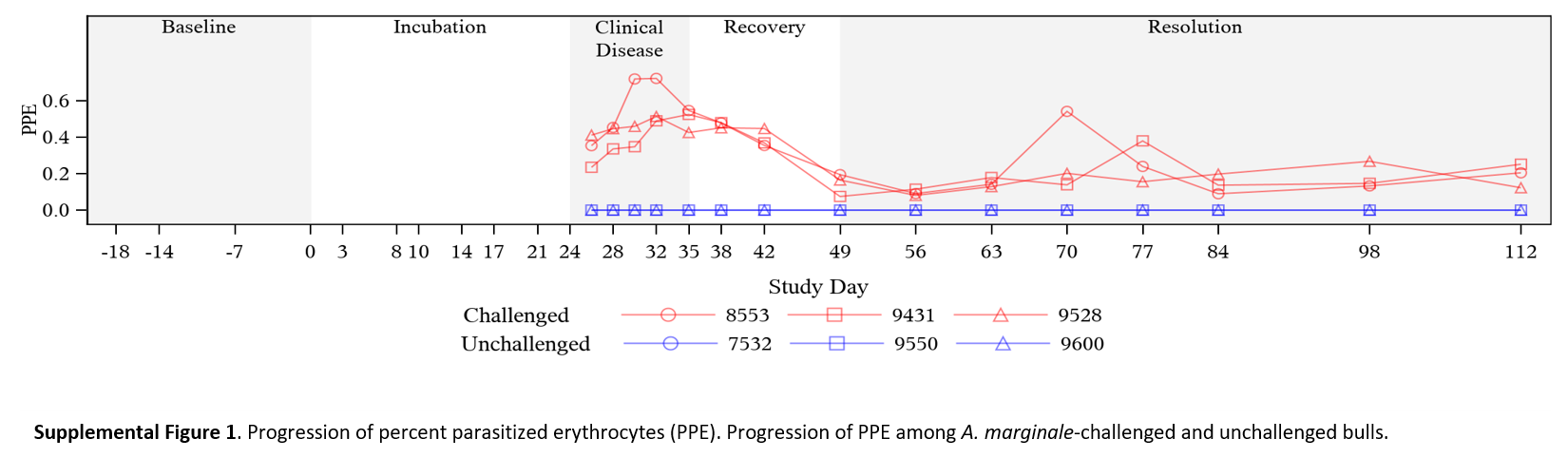

Supplement: Supplementary file 7 — Supplementary Material 7 [file 12917_2022_3470_MOESM7_ESM.png]
