# Supplementary material for: Satisfactory breeding potential is transiently eliminated in beef bulls with clinical anaplasmosis
Source: BMC Vet Res. 2022 Oct 29;18:381. doi: 10.1186/s12917-022-03470-7 (PMC9617051; doi:10.1186/s12917-022-03470-7)
Supplement: Supplementary file 4 — Supplementary Material 4 [file 12917_2022_3470_MOESM4_ESM.docx]

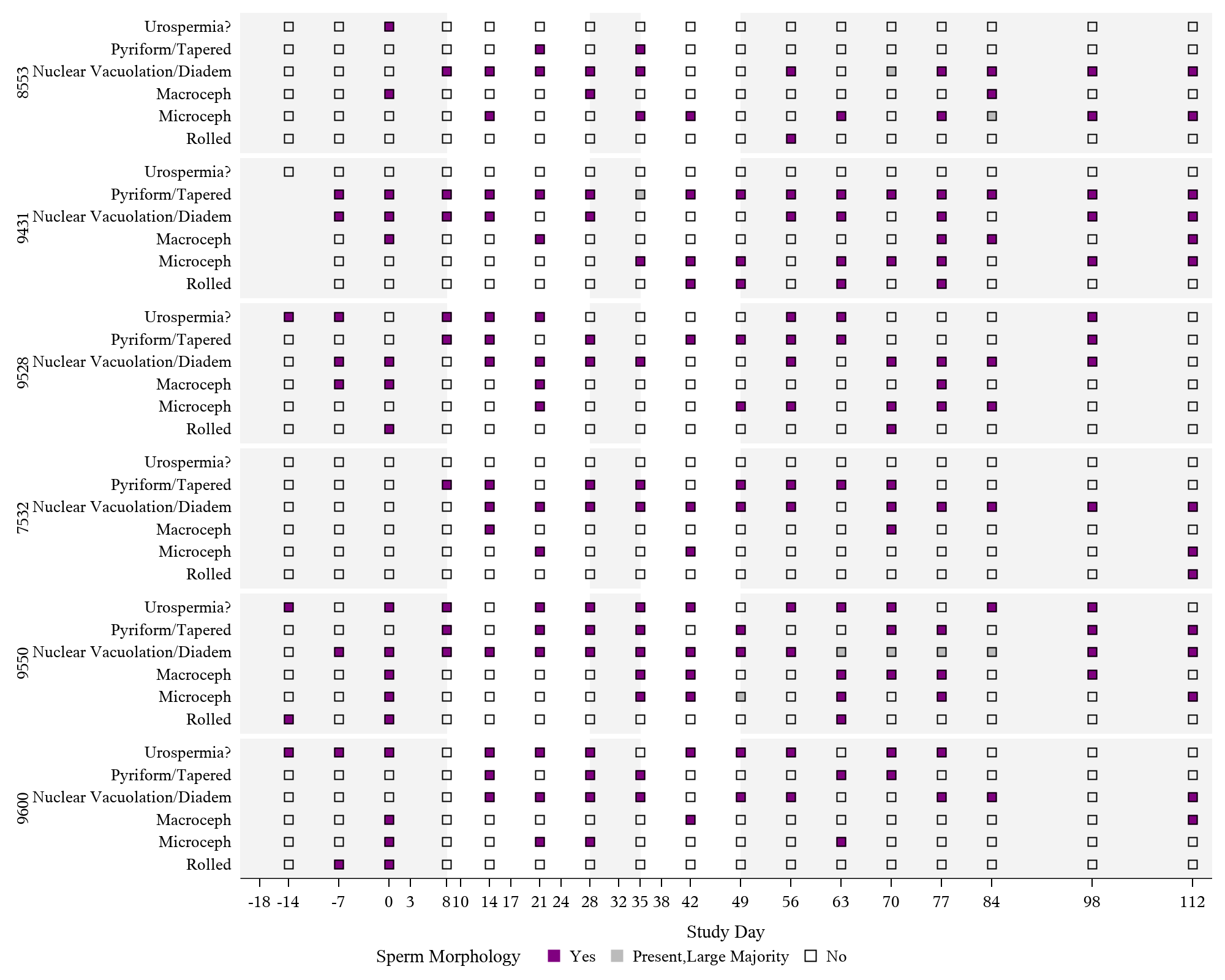


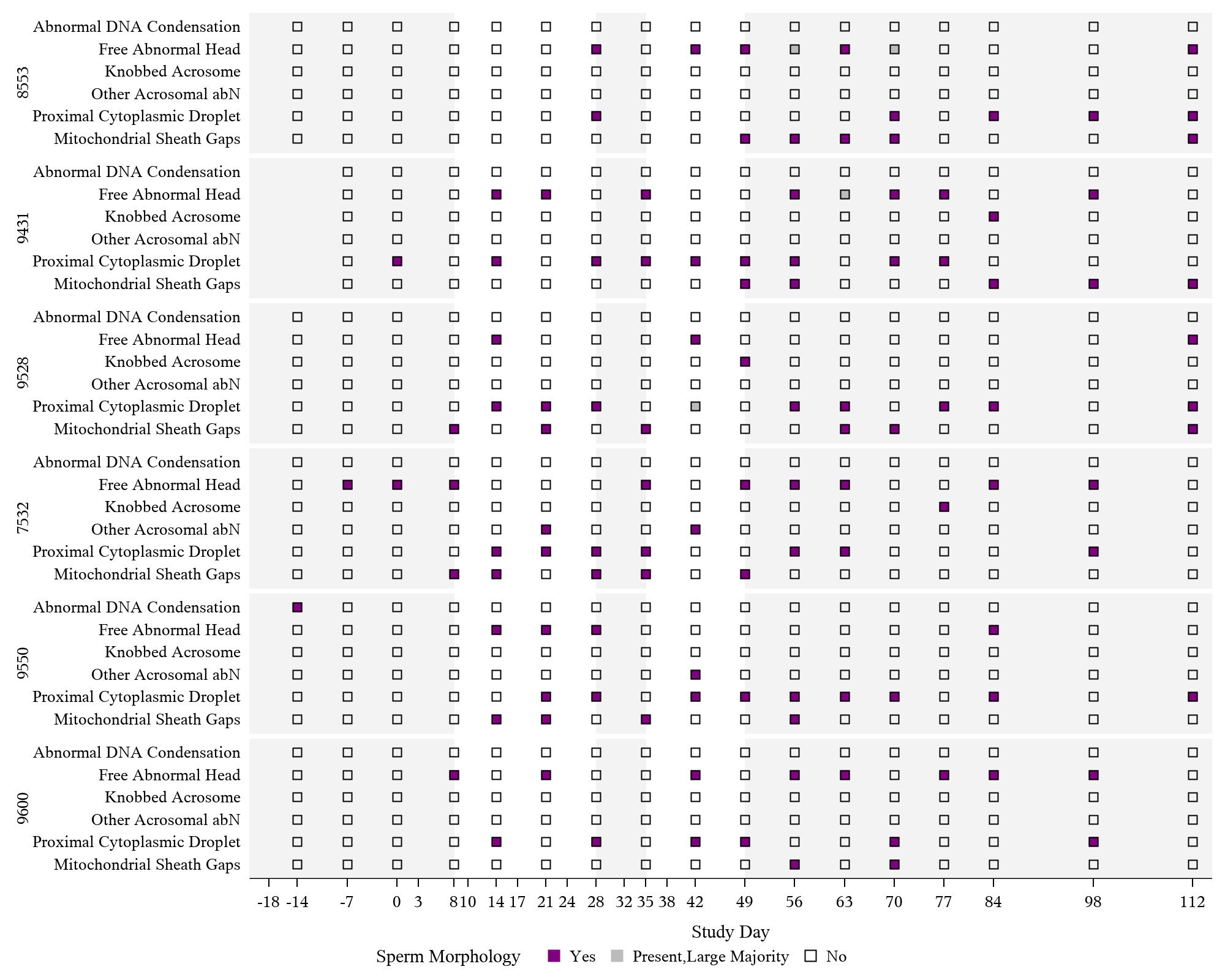


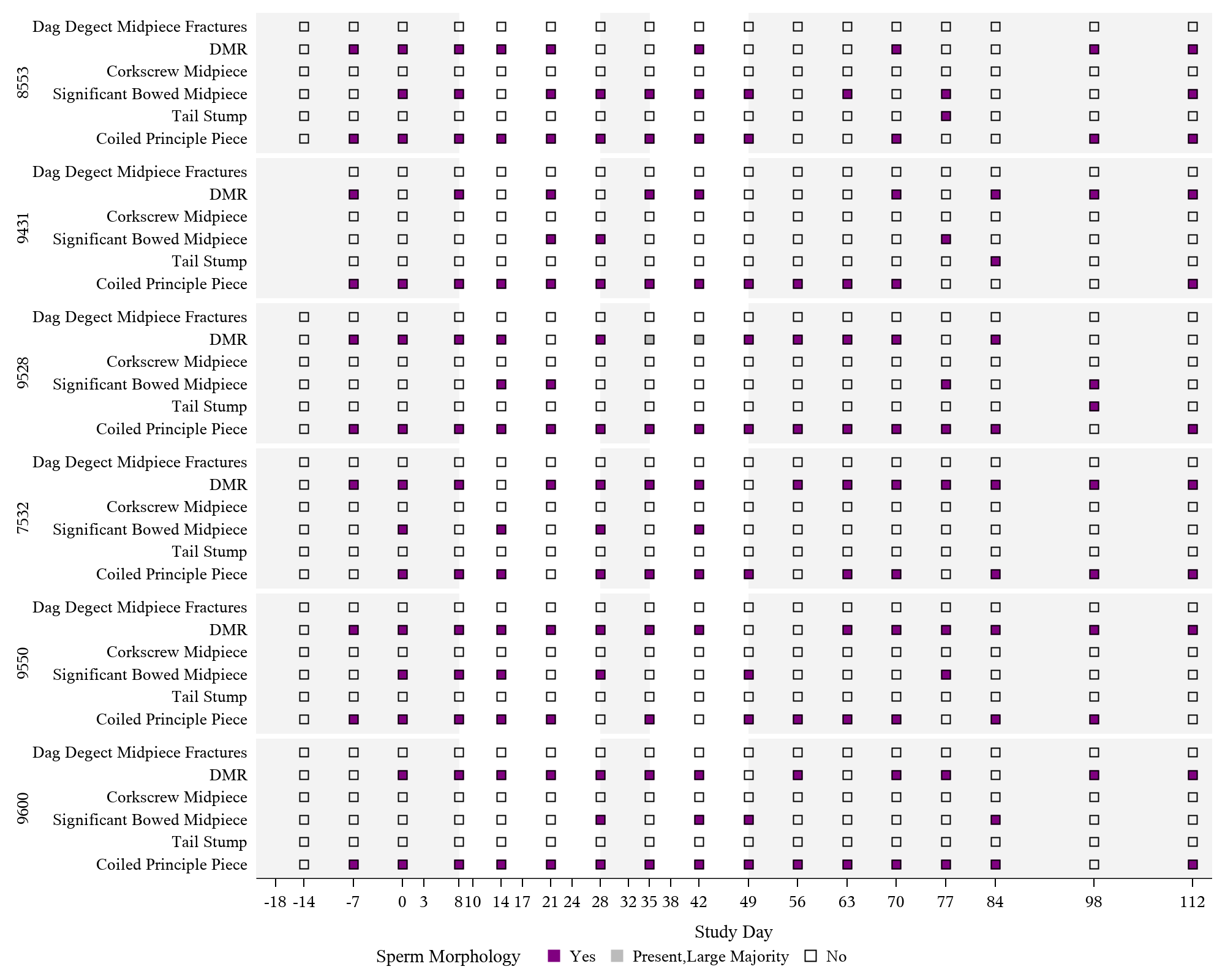


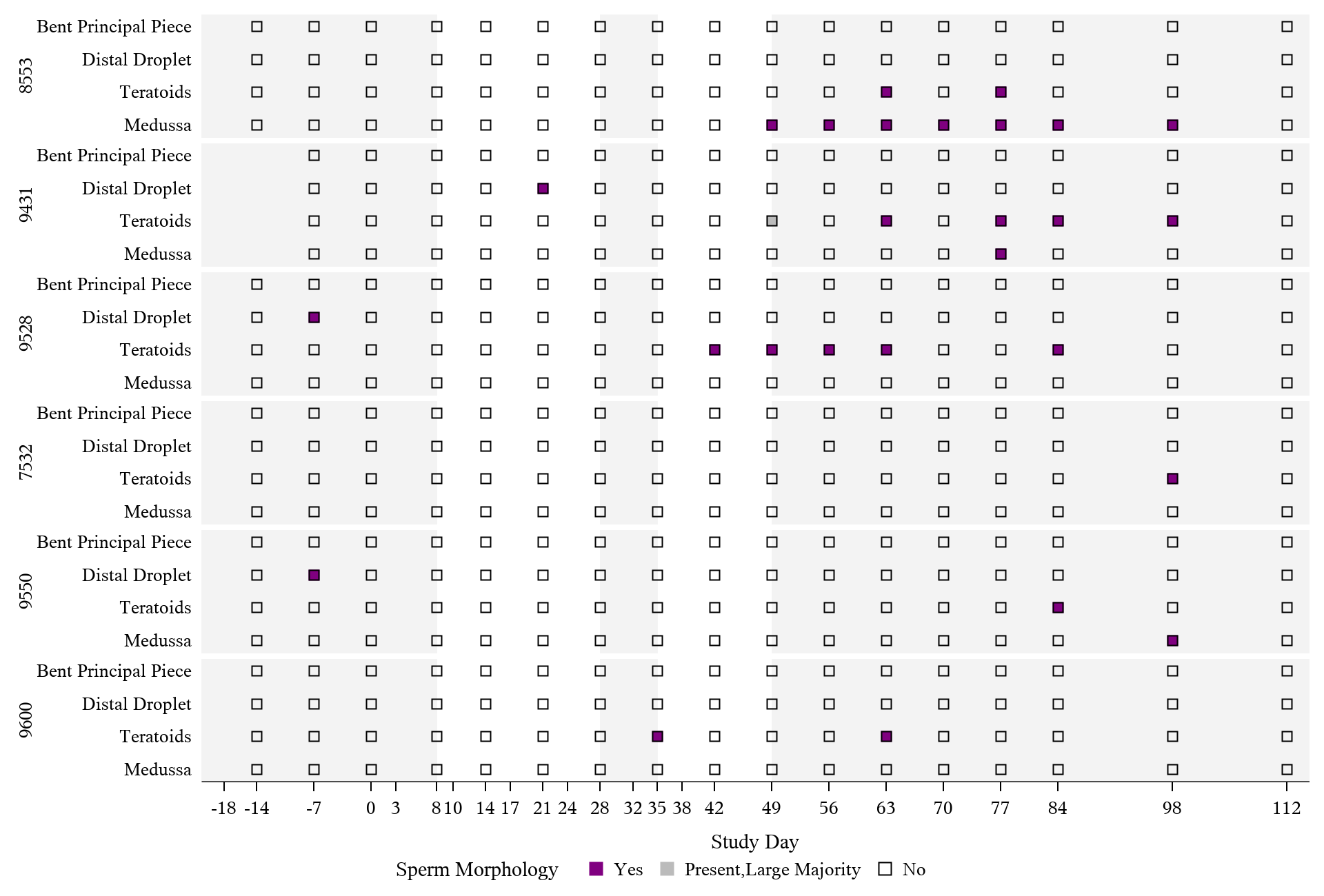


**Supplemental Figure 2**. Occurrence of specific sperm morphology abnormalities observed in individual *A. marginale*-challenged and unchallenged bulls. Purple shading indicates the abnormality was present; no shading indicates the abnormality was not present; and gray shading indicates the abnormality was present in abundance. Bulls 8553, 9431, and 9528 were challenged with *A. marginale* and bulls 7532, 9550, 9600 served as unchallenged controls.
