# Supplementary material for: Satisfactory breeding potential is transiently eliminated in beef bulls with clinical anaplasmosis
Source: BMC Vet Res. 2022 Oct 29;18:381. doi: 10.1186/s12917-022-03470-7 (PMC9617051; doi:10.1186/s12917-022-03470-7)
Supplement: Supplementary file 8 — Supplementary Material 8 [file 12917_2022_3470_MOESM8_ESM.docx]

**Additional Files Legends**

**Supplemental Figure 1. Progression of percent parasitized erythrocytes (PPE).** Progression of PPE among *A. marginale*-challenged and unchallenged bulls.

**Supplemental Figure 2**. **Occurrence of specific sperm morphology abnormalities observed in individual *A. marginale*-challenged and unchallenged bulls.** Purple shading indicates the abnormality was present; no shading indicates the abnormality was not present; and gray shading indicates the abnormality was present in abundance. Bulls 8553, 9431, and 9528 were challenged with *A. marginale* and bulls 7532, 9550, 9600 served as unchallenged controls.

**Supplemental Table 1. Summary statistics for blood parameters associated with *A. marginale* infection.** Mean, minimum and maximum packed cell volume (PCV), bacteremia (*A. marginale* (*Am*)/mL blood), and percent parasitized erythrocytes (PPE) among *A. marginale*-challenged and unchallenged bulls.

**Supplemental Table 2**: **Summary statistics for bull rectal temperatures.** Mean, minimum and maximum body temperatures (°C) among *A. marginale*-challenged and unchallenged bulls. Ambient outdoor temperature (°C) is also included.

**Supplemental Table 3**. **Summary statistics for bull body condition scores (BCS) and scrotal circumference throughout a course of clinical anaplasmosis.** Mean, minimum and maximum BCS and scrotal circumference (cm) among *A. marginale*-challenged and unchallenged bulls.

**Supplemental Table 4**. **Sperm progressive motility results.** Number of *A. marginale*-challenged and unchallenged bulls with a satisfactory percentage (>30%) of sperm with progressive motility; and, summary statistics (mean, minimum, maximum) of sperm with progressive motility.

**Supplemental Table 5**. **Summary statistics for normal morphology sperm.** Mean, minimum, and maximum percent of sperm with normal morphology among *A.* *marginale*-challenged and unchallenged bulls.

**Supplemental Table 6**. **Summary statistics for observed abnormal sperm morphology categories.** Mean, minimum, and maximum of abnormal morphology sperm categories among *A.* *marginale*-challenged and unchallenged bulls.
